# The Impact of Community Mental Health Programs for Australian Youth: A Systematic Review

**DOI:** 10.1007/s10567-022-00384-6

**Published:** 2022-02-16

**Authors:** Melissa Savaglio, Renee O’Donnell, Kostas Hatzikiriakidis, Dave Vicary, Helen Skouteris

**Affiliations:** 1 Health and Social Care Unit, School of Public Health and Preventive Medicine, Monash University, 553 St Kilda Road, Melbourne, VIC 3004 Australia; 2 Baptcare, Melbourne, Australia; 3 Warwick Business School, University of Warwick, Coventry, UK

**Keywords:** Youth, Mental health, Community-based programs, Systematic review

## Abstract

Australia has undergone significant youth mental health reform over the past 10 years, leading to numerous studies examining the effects of community-based mental health care programs for Australian youth. However, no synthesis of this literature currently exists. Therefore, this systematic review aimed to: (1) describe the types of community-based mental health programs that have been delivered to Australian youth in the past 10 years; and (2) examine their impact in improving young people’s mental health symptomology and psychosocial functioning. A systematic search of the peer-reviewed literature was conducted. Studies were included if they evaluated the extent to which such programs improved mental health symptomology (e.g., depression, anxiety, substance use) and/or psychosocial outcomes (e.g., social functioning, school engagement, employment) for Australian youth aged 10–25 years. Thirty-seven studies were included. Four types of community-based youth mental health care programs were identified: therapy (*n* = 16), case management (*n* = 9), integrated ‘one-stop-shop’ (*n* = 6) and lifestyle (*n* = 6) programs. The majority of therapeutic programs were effective in reducing mental health symptomology. Case management and integrated approaches consistently yielded significant improvements in both symptomology and psychosocial outcomes. Lifestyle programs were effective in alleviating depressive symptoms, but inconclusive for other outcomes. This review provides support for youth-friendly, systemic, multidisciplinary and integrated assertive outreach models of community mental health care to improve outcomes for young Australians experiencing mental health concerns. Several recommendations for future research are provided to strengthen the local evidence-base supporting community mental health programs to ultimately enhance young people’s life trajectory.

**Supplementary Information:**

The online version contains supplementary material available at 10.1007/s10567-022-00384-6.

## Introduction

### Youth Mental Health

The prevalence of mental health disorders (i.e., health conditions that are characterized by significant changes or disturbances in emotion, thinking, or behavior, American Psychiatric Association, 2020) among young people is a global health challenge (World Health Organisation [WHO], 2020). Recent estimates indicate that the worldwide-pooled prevalence of any mental disorder diagnosis among young people (defined herein as youth aged between 10 and 25 years old) is 10–20% (WHO, 2020). Anxiety is the most common mental health concern among 12–17-year-olds, and depression is one of the leading causes of illness among 10–19-year-olds (WHO, 2020). Specifically, approximately 13%, 16%, and 20% of adolescents from the United Kingdom, Europe, and the United States of America (USA), respectively, have a diagnosed mental health disorder (Unicef, 2021). Relatedly, suicide is the leading cause of death for adolescents in eastern Europe and central Asia, and the second leading cause in western Europe and the USA (Unicef, 2021). Undoubtedly, the global impact of the COVID-19 pandemic has further exacerbated the mental health of young people across the world; the presence of psychological distress among adolescents has more than doubled (Organisation for Economic Co-operation and Development, 2019). As the onset of approximately 80% of mental disorders tend to occur before the age of 25 (Caspi et al., 2020), youth mental health must maintain a global priority.

In Australia, approximately 14% of Australian youth experience a mental disorder each year (Australian Institute of Health & Welfare, 2020). Psychological distress among youth aged 15–19 continues to increase, from 19% in 2012 to 25% in 2019 (Hall et al., 2019) and suicide is the leading cause of death among 15–24-year-olds (AIHW, 2020). The prevalence of mental health concerns is consistently higher among particularly vulnerable groups of Australian youth, including young people living in out-of-home care (i.e., 3.8 times more likely to experience emotional and behavior concerns, Tarren-Sweeney, 2018), youth that identify as LGBTQIA + 1(i.e., five times more likely to attempt suicide, LGBTIQ + Health Australia, 2021), and Aboriginal and/or Torres Strait Islander youth (i.e., twice as likely to experience mental health concerns and four times the rate of suicide, Australian Bureau of Statistics, 2016), often stemming from the impacts of intergenerational trauma, colonization, dispossession, and marginalization. These figures have further increased given the devastating impact of the COVID-19 pandemic and associated lockdowns upon Australian young people (AIHW, 2021).

These high rates of mental health concerns, both locally and internationally, are concerning given their adverse personal, community, societal, economic, and intergenerational impact on young people. Particularly, youth with moderate to severe mental health concerns are also likely to experience significant impairments in psychosocial functioning, including social exclusion or poor social functioning (Gardner et al., 2019), reduced academic performance and school disengagement (Agnafors et al., 2020), lack of engagement in employment or community (Evensen et al., 2017), crime victimization and involvement in the juvenile justice system (Purcell & Harrigan, 2017), poor physical health (Curtis et al., 2016), persistent social disadvantage (Adriaanse et al., 2014), difficult family circumstances (Tarren-Sweeney, 2018), and intergenerational trauma and marginalization (Sapiro & Ward, 2020). These psychosocial factors may particularly account for the overrepresentation of mental health concerns among vulnerable population groups, such as Aboriginal youth. Finally, the presence of mental health concerns during youth has been well established as a pre-cursor for serious mental illness in adulthood, which supports repeated calls for greater investment into early intervention (McGorry & Mei, 2018).

### Barriers to Mental Health Support

Despite the rising prevalence of mental health concerns among youth, mental health service access and engagement among young people worldwide remains low, ranging from approximately 20–45% (Costello et al., 2014; Rocha et al., 2015). Globally, the gap between need and access to mental health care for youth is larger than any other age group (Radez et al., 2021). In Australia, it is estimated that at least 50–60% of youth are not receiving the treatment they require (AIHW, 2020). Several, ‘personal-level’ barriers among youth, such as perceived stigma, negative beliefs toward mental health services, cultural dissonance, and poor mental health literacy may contribute to this treatment gap (Radez et al., 2021; Velasco et al., 2020). There are also pronounced system-level barriers in reducing help-seeking behavior and access to mental health services, such as a lack of public awareness about available mental health services that are appropriate for young people, long waiting times, significant costs to access services, lack of transportation options to center-based support, limited flexibility of available appointment times (e.g., during school hours), and a lack of self-referral options for young people who are hesitant to approach a primary care provider (Anderson et al., 2017; Radez et al., 2021). Clearly, significant attention needs to be attributed to reducing this treatment gap and successfully engaging youth into mental health services to improve their wellbeing, long-term.

### Community-Based Mental Health Care

Mental health services delivered in the community, as opposed to mental health services that are delivered in acute health care settings (e.g., inpatient psychiatric care), residential treatment centers, or specialist mental health clinics, have been proposed as one viable, alternative method to address these barriers (Kwok et al., 2016; Rosen et al., 2010). This type of mental health care is embedded in the local neighborhood or community, delivered within the client’s existing natural environment, such as homes, workplaces/schools/universities, community centers, community-based organizations, community mental health clinics, recreational centers, and local youth points or local community outreach areas (i.e., parks). Community-based models of youth mental health care seek to increase accessibility, engagement, flexibility, and streamlining of support. Community mental health support is usually provided at a very low- or no-cost for the client, and young people are either proactively engaged in their own environment (i.e., at home) and/or supported to navigate referral pathways (Hetrick et al., 2017; Mantzouranis et al., 2019; Vijverberg et al., 2017).

Internationally, community-based approaches to youth mental health care have become widely adopted to facilitate increased service accessibility and improvements in wellbeing. Specific examples of well-established international community mental health care models include Jigsaw, an early intervention youth mental health service for youth aged 12–25 years that is implemented in Ireland (O’Keefe et al., 2015), Youthspace for youth aged 16–25 years in England (Vyas et al., 2015), and Foundry—an integrated health and social service for 16–24 year olds in Canada (Hetrick et al., 2017). A number of systematic reviews and meta-analyses have synthesized the effectiveness of international community-based programs in promoting the wellbeing of youth, observing improvements in mental health symptomology, self-esteem and confidence, substance use, behavioral outcomes, cognitive functioning, and social functioning (Farahmand et al., 2012; Garcia-Poole et al., 2019; Settipani et al., 2019; Vijverberg et al., 2017). Specifically, a meta-analysis by Farahmand et al. (2012) identified an overall moderate effect of community-based mental and behavioral programs on the health of low-income young people (predominantly from the USA), including decreased symptoms of depression, antisocial behavior, and improved interpersonal skills and physical health. Further, community-based group programs for adolescents with problematic behaviors were found to enhance their prosocial behavior and positive development, including social skills, empowerment, and self-esteem (Garcia-Poole et al., 2019). Significant improvements in psychological distress and psychosocial functioning have also been found for the integrated community-based programs from the UK where multidisciplinary support is provided at the one place, such as Jigsaw and Youthspace (Settipani et al., 2019). Finally, there are promising findings supporting youth assertive community treatment—multidisciplinary outreach support provided to young people in their own environment—in reducing the severity of psychiatric symptoms, improving general functioning and reducing psychiatric hospital admissions with varied effect sizes (Vijverberg et al., 2017). However, previous international syntheses have included either limited or no Australian literature, despite the existence of numerous local studies. Therefore, the evidence-base of such programs delivered in the Australian context remains underrepresented.

In Australia, significant attention has been directed toward mental health service reform in the recent decade to better address the mental health needs of young people (Malla et al., 2016). This has led to the recent paradigm shift that has placed the core focus of mental health service delivery on early intervention in the community (McGorry & Mei, 2018). Indeed, the recent national Inquiry into Mental Health concluded that increased accessibility to appropriate community-based support was required to reduce barriers that perpetuate the identified treatment gap of young people with mental health concerns (Commonwealth of Australia, 2020). One local example is Headspace—a national community-based and center-based mental health care model for you aged 12–25 years informed by the guiding principles of youth mental health services, with particular emphasis on embodying a holistic youth-friendly approach to promote inclusiveness, empowerment, and development (Hughes et al., 2016; Rickwood et al., 2019). Recent studies examining the effectiveness of Headspace have found significant reductions in psychological distress and improvements in psychosocial functioning (Bassilios et al., 2017; Rickwood et al., 2015). However, it has been noted that 40% of clients that present to Headspace are either too complex or severe to benefit from this entry level model of care (McGorry & Mei, 2018), and that numerous young people still face numerous barriers to accessing and engaging with such center-based support (e.g., outreach approaches are needed). Therefore, there is a need for more community-based models of mental health care that effectively meet the needs of young people with more severe mental health presentations and complex psychosocial needs (Commonwealth of Australia, 2020).

### Rationale for this Review

Despite the promise of community-based mental health care programs in supporting young people, the literature surrounding their evaluation in the Australian context has yet to be synthesized. While several community-based mental health care programs have been implemented across Australia, this lack of synthesis means that the type, quality, and size of the evidence-base is unknown. Synthesizing this literature is necessary to provide an overview of the current state of the community mental health sector and provide recommendations for future research. It may also contribute to a more comprehensive and holistic international understanding of youth community mental health programs, with Australia underrepresented in this area of research to date. This review is ultimately necessary to ensure that such programs are appropriately meeting the unique needs of youth, and to achieve further improvements in wellbeing. Therefore, the aim of this review was to: (1) describe the types of community-based mental health programs that have been delivered to Australian youth in the past 10 years; and (2) examine their impact in improving young people’s mental health symptomology and psychosocial functioning.

## Method

### Design and Protocol Registration

A systematic review was conducted, following the Preferred Reporting Items for Systematic Reviews and Meta-Analyses (PRISMA) statement guidelines (Page et al., 2021). This review protocol was registered with PROSPERO, an international database of prospectively registered systematic reviews in health and social care (Registration ID: CRD42020194043).

### Search Strategy

A systematic search of the empirical literature was conducted in June 2021 for studies that had evaluated the impact of community-based mental health programs for youth in Australia. Four electronic databases were searched, including: PsycINFO, PsycArticles, MEDLINE, and CINAHL plus. These databases were chosen to capture literature in the field of psychology as well as broader health (inclusive of mental health). Various combinations of the following keywords were used in the search: “*youth*”, “*mental health*”, and *“program”* (see Supplementary Table 1 for the full search strategy). The database selection and search terms were developed in consultation with a librarian, and terms were also drawn from similar international systematic reviews examining youth mental health (e.g., Kowk et al., 2016; Vijverberg et al., 2017). The search strategy also incorporated medical subject heading (MESH) search terms and keywords, which were customized to each database as needed. The reference lists and citations for included articles were also examined.

### Inclusion and Exclusion Criteria

Studies were included in the review if they met the following inclusion criteria: (1) the study participants were young people aged between 10 and 25 years^2^ experiencing mental health concerns; (2) the study was conducted in Australia; (3) the study quantitatively evaluated the impact of a mental health program for youth that was delivered within the community (e.g., delivered in homes, community centers, rather than residential or inpatient services, etc.); (4) study outcomes included mental health symptomology (e.g., symptoms of depression, anxiety, psychosis, etc.) and/or psychosocial functioning (e.g., social functioning, education, employment, etc.); (5) the study employed a quantitative evaluation study design (i.e., randomized controlled trial, quasi-experimental, or pre-post study design); and (6) the study was published in English from January 2011 to June 2021 inclusive. Given the relatively recent paradigm shift toward community-based mental health care for youth in Australia (Kwok et al., 2016; Rosen et al., 2010), studies were restricted to the past 10 years to provide a snapshot of the most current empirical evidence regarding the impact of interventions to inform current practice. Young people encompassed individuals aged from 10 to 25 years to align with well-established international and local youth mental health services that provide support to young people aged up to 25 years to improve continuity of mental health care for transition-age youth, rather than discontinuing support at 18 years of age (Nguyen et al., 2017; Rickwood et al., 2015).

Studies were excluded if: (1) they did not examine the impact of the intervention on the young people themselves (e.g., implementation outcomes, cost-effectiveness, only parental outcomes); (2) they examined young people with a sole primary diagnosis of a neurodevelopmental disorder (i.e., autism spectrum disorder, attention deficit/hyperactivity disorder, learning disability, or intellectual disability); (3) the young people were considered “at-risk” of developing mental health concerns (i.e., prevention programs); and (3) if the intervention was school-based or a smartphone or technological intervention (e.g., mobile phone applications) as these have already been extensively reviewed (Das et al., 2016; Hollis et al., 2016) and were not considered under the community-based scope of this review due to their inherent differences in delivery. Specifically, Australian school-based approaches have been recently synthesized and international research has consistently examined programs delivered within the formal school setting and community-based mental health programs separately (Das et al., 2016; Dray et al., 2017).

### Study Selection

Two researchers independently screened and excluded studies based on titles and abstracts. For articles not excluded, the full-text versions were sourced and assessed for inclusion by the same two researchers. The interrater agreement, calculated by the proportion of studies that were given the same rating by the two researchers during the title and abstract stage and full-text stage, was 0.94 and 0.97, respectively. Any inconsistencies were resolved by consulting a third independent researcher.

### Data Extraction and Synthesis

Summary tables were created to extract data from the included studies (see Supplementary Tables 2 and 3). One researcher extracted data from all included studies, with approximately 30% (*n* = 13) cross-checked for accuracy. There was 100% agreement in data extracted so no additional changes were made. Data extracted included: state where the study was conducted; study design; sample size; participant diagnosis, age, and gender; intervention description, duration, and frequency; assessment time points; outcomes; measures; and findings. As there was significant heterogeneity across studies in terms of study design, outcomes, measures, and reported data, a meta-analysis was not possible (Cuijpers et al., 2017). Instead, the findings were categorized according to intervention type and their impact was detailed descriptively by study design.

### Quality Assessment

The methodological quality assessment of included studies was conducted independently by two researchers using two measures, depending on study design; the National Institute of Health (NIH) Quality Assessment Tool of Controlled Intervention Studies and the NIH Quality Assessment Tool for Before-After (Pre-Post) Studies (NIH, 2021). These scales rate key aspects of methodological quality (i.e., blinding, attrition rate, sample size and power, outcome measures, intervention adherence, etc.) as “yes,” “no,” or “not reported”. Studies that scored “yes” for at least 75% of their assessment criteria were categorized as “high” quality due to low risk of bias, studies that fulfilled 50% to 74% of the criteria were classified as “medium” quality, and studies that scored “yes” for less than 50% of the criteria were considered “low” quality due to high risk of bias. Consensus was achieved through a cooperative discussion between the two researchers, where the interrater agreement (proportion of agreed ratings) was 0.96. No studies were excluded from the synthesis based on their quality assessment outcome to ensure that the findings of all relevant literature in this area were captured to provide a holistic snapshot of the evidence-base in this preliminary review of community-based mental health programs for Australian youth.

## Results

### Search Yield

**Fig. 1:**
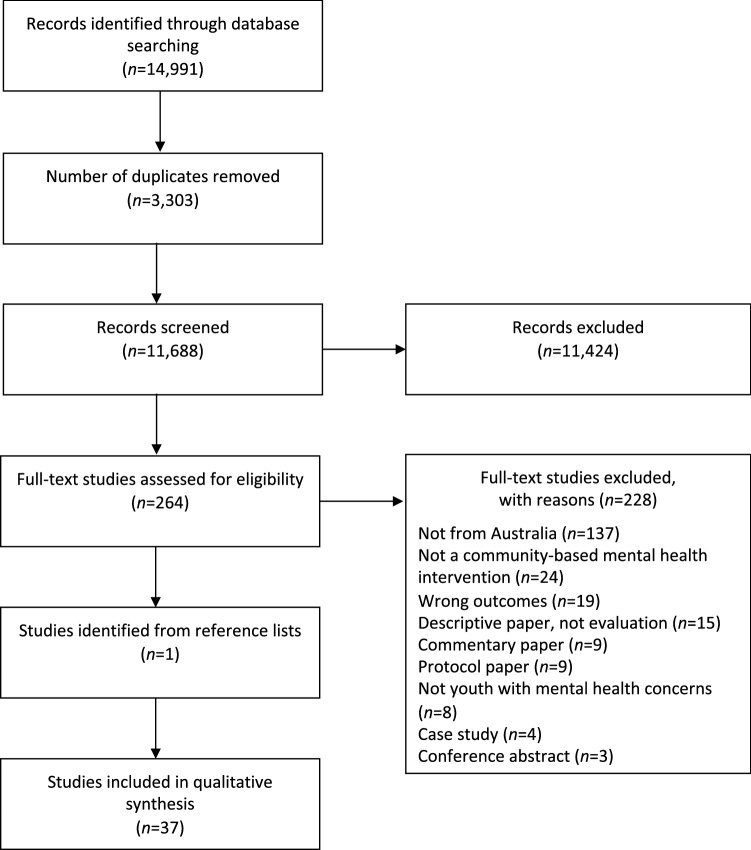
PRISMA flowchart of study selection

The stages of study selection are summarized in the PRISMA flowchart presented in Fig. 1. The search of the four electronic databases identified a total of 14,991 studies. After the removal of 3303 duplicates, 11,688 studies were screened for eligibility at the title and abstract stage, followed by 264 full-text studies. During the full-text stage, 228 studies were excluded; the predominant reason for exclusion was that the study was not conducted in Australia (*n* = 137).^3^ A total of 36 studies were deemed eligible for inclusion, and one additional study was found by consulting their reference lists. Therefore, a total of 37 studies were included in this review.

### Quality of Studies

A summary of the quality assessment for the 37 studies included in this review is presented in Supplementary Table 4. There were 24 pre-post studies and 13 controlled studies (i.e., eight randomized controlled trials and five quasi-experimental studies without random allocation to groups). The majority of the controlled studies (6/13, 46%), were classified as medium quality, four studies (31%) were assessed as high quality and three (23%) studies were considered low quality due to high risk of bias. Randomization was generally adequate across studies, with groups similar at baseline in 10 studies (77%). Seven controlled studies (54%) had sufficiently large sample sizes to detect significant differences with 80% power. Seven studies (54%) reported rates of attrition of less than 20% at the endpoint of the study. Limitations included a lack of blinding of participants or providers in 12 studies (92%) and outcome assessors in 8 studies (62%), and a lack of reporting of intervention adherence (69%). Outcome measures were assessed using valid and reliable measures.

The 24 pre-post studies were predominantly of medium quality (15/24, 63%), with six studies (25%) classified as low quality due to high risk of bias, and only three studies (12%) considered high quality. Eligibility criteria were often prespecified and clearly described in 16 studies (67%), and appropriate outcome measures and statistical methods were utilized to measure pre-post changes. However, studies consistently did not blind outcome assessors (23/24, 96%), and sample sizes were generally not large enough to provide confidence in the findings (14/24, 58%) or were not adequately representative of the target population (13/24, 54%).

### Summary of Studies

A summary of each study’s design, participant characteristics, and intervention content is presented in Supplementary Table 2. All studies were conducted across various states and territories in Australia, with the majority from Victoria (*n* = 19), followed by New South Wales (*n* = 6, Conrad et al., 2017; Curtis et al., 2016; Howe et al., 2017; Hudson et al., 2015; Nasstasia et al., 2017, 2019), Queensland (*n* = 5, Daubney et al., 2021; Edwards et al., 2018; Farrell et al., 2012; Klag et al., 2016; Tan & Martin, 2015), Western Australia (*n* = 3, Goel et al., 2021; Porter & Nuntavisit, 2016; Sabbioni et al., 2018), and Tasmania (*n* = 1, Westwater et al., 2020). Two studies were conducted nationally (Bassilios et al., 2017; Rickwood et al., 2015), and the final study collected data from three states (VIC, NSW, and QLD, Hall et al., 2021).

### Participant Characteristics

The mean sample size across the 37 studies was 194 (SD = 359), ranging from 4 to 1997 persons. This excludes the two national studies with larger sample sizes of 37,493 (Bassilios et al., 2017) and 20,034 (Rickwood et al., 2015). The proportion of young people who identified as female across the studies was 58%. Participants were aged 18 years or younger in sixteen of the 37 studies (43%), 15 studies (41%) included young people aged between 15 and 25 years, and the remaining six studies (16%) included 12–25-year-olds. The average age across all studies was 16.52 years (*SD* = 3.26).^4^ The majority of studies (*n* = 23) included a sample of young people with varied mental health concerns (e.g., depression, anxiety, psychosis, etc.). The remaining studies examined young people with the same primary diagnosis, including first-episode psychosis (*n* = 5, Brewer et al., 2015; Conrad et al., 2017; Curtis et al., 2016; Killackey et al., 2019; McGuire et al., 2021), depressive disorders (*n* = 5, Hayes et al., 2011; Hides et al., 2011; Nasstasia et al., 2017, 2019; Poole et al., 2018), anxiety disorders (*n* = 1, Hudson et al., 2015), obsessive–compulsive disorder (*n* = 1, Farrell et al., 2012), and anorexia (*n* = 1, Green et al., 2015). The diagnosis severity of fourteen of the 37 samples (38%) was considered “severe” based on clinical measures of symptom severity with established cut-off scores or diagnostic status following clinical diagnostic interviews. These participants experienced complex, persistent, high risk or severe mental health presentations, as defined in the DSM-5 (APA, 2013). There was also a high rate of comorbidity across the studies, with an average of 64% of young people experiencing more than one mental health concern, most commonly comorbid depression and substance use.

Less than half of the studies (*n* = 14) reported the ethnicity of participants. Aboriginal and/or Torres Strait Islander young people were consistently underrepresented, with one study evaluating a program that was delivered specifically to young people who identified as Aboriginal (Sabbioni et al., 2018). Only eight studies described the cultural and linguistic diversity of the sample, defined as being born in non-English speaking countries (*n* = 7, Hall et al., 2021; Hayes et al., 2011; Killackey et al., 2017, 2019; McGuire et al., 2021; Pearce et al., 2020; Sabbioni et al., 2018) or do not speak English at home (*n* = 1, Kehoe et al., 2014), yet the specific countries were often not specified. There was also a lack of inclusion and reporting on the sexual and gender diversity of young people in the reviewed studies (*n* = 4): two studies each included two young people who identified as non-binary or gender queer (i.e., do not identify as male or female, Hall et al., 2021; Hickey et al., 2020); one study reported that 3% of the sample identified as transgender or gender diverse (Goel et al., 2021); and 15% of young people identified as same-sex attracted or bisexual in Daubney et al.’ (2021) study. Only five studies discussed families’ socioeconomic status, encompassing parental income, educational attainment or employment, which were considered representative of the general population (Bassilios et al., 2017; Farrell et al., 2012; Havighurst et al., 2015; Hides et al., 2011; Kehoe et al., 2014). Two studies reported that the majority of young people (66% and 90%) were receiving government benefits (Hides et al., 2011; Killackey et al., 2019). Finally, one study assessed a program that was specifically tailored for youth living in in out-of-home care (Klag et al., 2016).

### Characteristics of Interventions

Interventions were delivered weekly for 4.5 months on average, (*M* = 18.17 weeks, SD = 17.34). However, there was significant variation in the duration and intensity of support across interventions depending on the young person’s needs, ranging from one session of family therapy (Hopkins et al., 2017; Westwater et al., 2020) to over 18 months of engagement (Klag et al., 2016). Frequency of contact varied, with up to three times a week or daily for the more intensive programs (Green et al., 2015; Porter et al., 2016; Schley et al., 2012). All interventions were delivered in community-based settings. Specifically, 13 out of 37 interventions were center-based, delivered at various community mental health clinics across the country (such as Headspace, Australia’s national youth mental health service). Four programs were conducted at a University (Farrell et al., 2012; Hudson et al., 2015; Nasstasia et al., 2017, 2019) while three programs were delivered at a local community center (Havighurst et al., 2015; Kehoe et al., 2014; Tan & Martin, 2015). Eight studies were conducted entirely via assertive outreach, predominantly in clients’ homes, or other community meeting points, such as parks, cafes, skateparks, or during transportation to various appointments to engage the young people within their naturalistic environment (Brewer et al., 2015; Chia et al., 2013; Daubney et al., 2021; Howe et al., 2017; Killackey et al., 2017; Porter & Nuntavisit, 2016; Sabbioni et al., 2018; Schley et al., 2012). The remaining nine programs were delivered across various types of community settings, including a combination of center-based and outreach locations (e.g., homes, Curtis et al., 2016; Conrad et al., 2017; Goel et al., 2021; Hall et al., 2021; Hides et al., 2011; Killackey et al., 2019; Pearce et al., 2020; Poole et al., 2018; Wagner et al., 2017).

Sixteen of the 37 interventions were implemented in a group format (i.e., group therapy or exercise programs). Ten interventions offered a parent component, where parents were either encouraged to attend group therapy sessions with the young person (e.g., Hudson et al., 2015), or received education, strategies and skill development to better support their child (e.g., Farrell et al., 2012). Interventions were predominantly delivered by psychologists (*n* = 14), a multidisciplinary team of health professionals (*n* = 13), including psychologists, psychiatrists, social workers, mental health nurses, clinical nurses, counselors, dieticians, and case managers, or one key mental health clinician (*n* = 5). The remaining programs were implemented by an exercise physiologist (*n* = 1, Pearce et al., 2020), personal trainer (*n* = 2, Nastassia et al., 2017, 2019), or vocational and education specialist (*n* = 2, Killackey et al., 2017, 2019). These programs that were not delivered by mental health specialists were still considered community-based mental health programs as they were described as such by the authors, and aimed to support and improve the mental health and psychosocial functioning of young people experiencing mental health concerns via community-based delivery.

### Intervention Content

Four different types of community-based mental health programs for youth were identified across the 37 studies: therapy (*n* = 16), case management (*n* = 9), integrated one-stop shop^5^ (*n* = 6), and lifestyle (*n* = 6) interventions. Detailed descriptions of each intervention are presented in Supplementary Table 2.

#### Therapy Programs

Sixteen of the 37 studies evaluated the impact of a therapy-based program for young people, predominantly delivered by a psychologist. Nine of the 16 studies implemented a manualized group therapy program, which included: family-based group Cognitive Behavioral Therapy (CBT) program for youth with obsessive compulsive disorder (OCD, Farrell et al., 2012); the Cool Kids program, which implemented CBT for youth with anxiety disorders (Hudson et al., 2015); origami and mindfulness-based art therapy group program (Edwards & Hegerty, 2018); Emotion Regulation and Impulse Control (ERIC) modularized CBT skills-based program (Hall et al., 2021); the Mindfulness and Compassion group program for young people experiencing psychosis (Hickey et al., 2020); the BEST MOOD program—a manualized eight-session family systems group therapy program (i.e., parent and young person sessions) to alleviate depressive symptoms (Poole et al., 2018); Tuning in to Teens, a six-session group therapy program specifically targeted to parents to help support their adolescents to understand and cope with their emotions in a positive way (Havighurst et al., 2015; Kehoe et al., 2014); and a five-session mindfulness-based group program called Taming the Adolescent Mind (Tan & Martin, 2015). Group programs were conducted weekly at local community centers or services and ranged in duration from one to three months.

Four studies evaluated individual therapy programs. Hides et al. (2011) evaluated individual cognitive behavioral therapy combined with motivational interviewing strategies to support youth with comorbid depression and substance use. Acceptance and Commitment Therapy (ACT) involved the use of acceptance and mindfulness-based strategies combined with commitment and behavior change strategies to alleviate symptoms among youth with moderate to severe depression (Hayes et al., 2011). Two studies evaluated brief intervention therapy—up to six sessions of cognitive behavioral-based psychological therapy among young people case managed by Child and Adolescent Mental Health Services (CAMHS, Wagner et al., 2017) or young people attending Headspace (Schley et al., 2018). Finally, two studies evaluated the benefit of a single family therapy session, where clinicians adopted a systemic approach in a one-off family session that aimed to provide clinical care to the young person and enable family members to provide ongoing support at home (Hopkins et al., 2017; Westwater et al., 2020). One study evaluated multi-systemic therapy, where therapists provided highly intensive home-based systemic treatment (i.e., three home visits per week), using a range of evidence-based therapy interventions (Porter & Nuntavisit, 2016).

#### Case Management Programs

Nine of the 37 studies employed a case management approach to youth community mental health care. Seven of these studies implemented an assertive outreach model of case management support, which involved a multidisciplinary team of clinicians (i.e., psychologists, social workers, clinical nurses, case managers, psychiatrists) who provided tailored wrap-around support to meet each young person’s individualized psychosocial needs via flexible service delivery and assertive outreach (i.e., home visits, ‘in-transit’, intensive or extended contact hours, and practical support, etc.). The specific names of each assertive outreach service and their further details are presented in Supplementary Table 2 (Brewer et al., 2015; Chia et al., 2013; Conrad et al., 2017; Daubney et al., 2021; Howe et al., 2017; Sabbioni et al., 2018; Schley et al., 2012). These programs are distinguished from traditional case management by smaller caseloads (1:5), longer duration (i.e., 10–18 months), higher frequency of client contact, greater care coordination, multidisciplinary support, and an emphasis on outreach, including the provision of in-home support, to engage youth in holistic assessment and treatment. The remaining two studies evaluated the effect of Individual Placement and Support for Education (IPSE) via a pre-post study (Killackey et al., 2017), followed by an RCT (Killackey et al., 2019). IPSE involved an education specialist supporting young people with severe mental health concerns to engage in employment, vocational training or education.

#### Integrated One-Stop-Shop Services

Six of the studies assessed an integrated ‘one-stop shop’ model of community mental health care, categorized by the provision of in-house or ‘hub-like’ multidisciplinary clinical and psychosocial support all available at the one location, without brokering services or supports. Two studies evaluated Headspace, which provided a range of in-house support for young people, including psychological (individual sessions with a psychologist and a broad range of group programs), physical and sexual health, alcohol and/or other drugs, and vocational services (Bassilios et al., 2017; Rickwood et al., 2015). Kennair et al. (2011) evaluated the Adolescent Day Program, an intensive community-based group program (3 × 5-h sessions per week) for young people case managed by CAMHS, which included skills-based work (i.e., social skills training, anger management), practical community outings, and a focus on the achievement of psychosocial goals. Klag et al. (2016) evaluated Evolve Therapeutic Services, a trauma-informed wrap-around model of care where a multidisciplinary team provides coordinated in-house therapeutic and behavioral supports to young people in out-of-home care. Similarly, youth received multidisciplinary in-house support (i.e., therapy, medication, and support with psychosocial stressors, such as housing, finances, schooling) from the Youth Community Assessment and Treatment Team (Goel et al., 2021). Finally, Green et al. (2015) evaluated the Butterfly Eating Disorder Day Program for young people with eating disorders, a multidisciplinary program comprising of cognitive behavioral group therapy five days a week, access to a key support worker to conduct case management depending on the broader psychosocial needs of each young person, as well individual, dietic, and family support.

#### Lifestyle Programs

The six remaining studies implemented lifestyle interventions focused on increasing engagement in healthy lifestyle behaviors, particularly physical activity (Curtis et al., 2016; McGuire et al., 2021; Nasstasia et al., 2017, 2019; Parker et al., 2016; Pearce et al., 2020). Such programs were considered within the scope of community-based mental health programs as they sought to improve mental health outcomes among youth in a community setting. Healthy Body Healthy Mind was a 12-week multi-modal exercise program facilitated by personal trainers who utilized motivational interviewing techniques to engage youth in physical activity and address barriers to ongoing engagement (Nasstasia et al., 2017, 2019). A six-week physical activity intervention underpinned by psychoeducation, behavioral activation, goal setting, and weekly monitoring was assessed by Parker et al. (2016). The Keeping the Body in Mind (KBIM) program was a multi-modal health-based intervention comprising tailored exercise programs, nutritional support, and wellness coaching delivered by a multidisciplinary team of allied health professionals (Curtis et al., 2016). An exercise physiology service embedded within standard youth community mental health care provided young people with an individualized gym program to follow and group exercise programs, such as yoga or boxing (Pearce et al., 2020). Similarly, McGuire et al. (2021) embedded a yoga program within an early intervention for psychosis service in which young people engaged in group yoga sessions focused on relaxation, grounding, and breathing techniques.

### Control Groups

Nine of the 13 controlled studies evaluated the intervention against treatment as usual (i.e., standard care). Type of standard care often depended on diagnosis, including case management combined with motivational interviewing for comorbid depression and substance misuse (Hides et al., 2011), standard cognitive behavioral therapy for depression (Hayes et al., 2011); and standard case management for varied moderate to severe mental health concerns, which included assessment, medication management, psychological treatment, and referrals to other services (Curtis et al., 2016; Kennair et al., 2011; Killackey et al., 2019; Tan & Martin, 2015; Wagner et al., 2017). Two studies employed an alternative comparison group; a Parenting Adolescents Support Training group program for parents was a comparison for the BEST MOOD program (Poole et al., 2018), and Allied Psychological Services, a primary care model of psychological treatment, was compared to Headspace (Bassilios et al., 2017). The control participants in the three remaining controlled studies did not receive any treatment (Havighurst et al., 2015; Kehoe et al., 2014; Nasstasia et al., 2019).

### Intervention Impact

The two broad outcomes assessed across all 37 studies were mental health symptomology (i.e., encompassed general symptom severity, psychological distress, depressive symptoms, anxiety symptoms, OCD symptoms, and substance use) and psychosocial functioning (i.e., encompassed social relationships and skills, engagement in education or employment, general functioning, and physical health). Mental health symptomology was most commonly assessed by the Health of the Nations Outcomes Scale for Children and Adolescents, followed by the Kessler Psychological Distress Scale or the Strengths and Difficulties Questionnaire. Psychosocial functioning was predominantly measured using the Children’s Global Assessment of Functioning or the Social and Occupational Functioning Assessment Scale. Due to the significant heterogeneity of the studies in terms of study design, intervention, outcomes, and measures, and lack of statistical reporting, meta-analysis was not feasible. Therefore, intervention impact was synthesized narratively by type of intervention, with consideration of the different study designs (pre-post and controlled studies). Further details, including effect sizes, are presented in Supplementary Table 3. Cohen’s *d*-test was used to report effect sizes, with the well-established benchmarks of 0.20, 0.50 and 0.80 used to indicate small, moderate, or large effects, respectively (Cohen, 1988).

#### Therapy Programs

Fifteen of the 16 therapy programs (*n* = 7 controlled, *n* = 8 pre-post) evaluated improvements in mental health symptomology, while only five studies (*n* = 4 pre-post, *n* = 1 controlled) assessed psychosocial functioning (Farrell et al., 2012; Hickey et al., 2020; Hides et al., 2011; Hopkins et al., 2017; Schley et al., 2018). Eleven of the 15 studies (8/8 pre-post, 3/7 controlled) found significant improvements in young people’s mental health symptoms following engagement in a therapy program, with variability in effect sizes ranging from very small (*d* = 0.14, Kehoe et al., 2014) to very large (*d* = 2.07, Hickey et al., 2020). Specific outcomes included significant reductions in general symptom severity, (*d* = 0.31–2.07, Hall et al., 2021; Havighurst et al., 2015; Hickey et al., 2020; Porter & Nuntavisit, 2016), depressive symptoms (*d* = 0.14–1.45, Hayes et al., 2011; Kehoe et al., 2014; Schley et al., 2018), anxiety (*d* = 0.46–0.86, Hudson et al., 2015; Kehoe et al., 2014; Schley et al., 2018; Westwater et al., 2020), OCD symptoms (*d* = 0.92, Farrell et al., 2012) and psychological distress (*d* = 0.75, Schley et al., 2018; Tan & Martin, 2015). In contrast, youth from the remaining four controlled studies who received cognitive behavioral therapy, family therapy, or brief intervention therapy did not achieve significantly better mental health outcomes than the control group (Hides et al., 2011; Edwards & Hegerty, 2018; Poole et al., 2018; Wagner et al., 2017). Finally, four of the five studies (all pre-post) found significant improvements in young people’s general and social functioning with moderate to large effect sizes following engagement in CBT for OCD (*d* = 0.69, Farrell et al., 2012), mindfulness and compassion therapy (*d* = 1.32, Hickey et al., 2020), single session therapy (Hopkins et al., 2017) and brief intervention therapy (*d* = 0.59, Schley et al., 2018). The controlled trial of CBT did not yield any significant improvements in psychosocial functioning between treatment groups (Hides et al., 2011).

#### Case Management Programs

Six of the nine case management interventions were evaluated by the extent of pre-post improvements in mental health symptomology, eight studies (*n* = 7 pre-post, *n* = 1 controlled) assessed psychosocial functioning, and four of nine studies (all pre-post) measured hospital admissions (Brewer et al., 2015; Chia et al., 2013; Conrad et al., 2017; Daubney et al., 2021). All six studies that assessed young people’s mental health symptomology, all via pre-post evaluation, yielded a significant reduction in general symptom severity with consistently large effect sizes (*d* = 0.80–1.37, Brewer et al., 2015; Conrad et al., 2017; Daubney et al., 2021; Howe et al., 2017; Killackey et al., 2017; Schley et al., 2012). Further, young people reported a medium to large reduction in suicidality (Daubney et al., 2021; Schley et al., 2012), and anxiety and depressive disorders (Conrad et al., 2017) following assertive case management. All eight studies (*n* = 7 pre-post, *n* = 1 controlled) that evaluated young people’s psychosocial wellbeing found significant improvements following assertive outreach. Specifically, Brewer et al. (2015) observed improvements in social functioning among youth who received intensive case management (*d* = 0.38). Further, 95% of youth who received Individual Placement and Support were engaged in education at follow-up (Killackey et al., 2017), and had significantly greater odds of being employed compared to control participants (Killackey et al., 2019). All four pre-post studies that examined hospitalizations observed a significant reduction in the rate of admissions following case management (Brewer et al., 2015; Chia et al., 2013; Conrad et al., 2017; Daubney et al., 2021). Daubney et al. (2021) saw increased emergency department visits at follow-up, attributed to increased monitoring of adolescents engaged in the outreach service.

#### Integrated One-Stop-Shop Services

All six studies (*n* = 3 controlled, *n* = 3 pre-post) that evaluated integrated community mental health interventions assessed changes in mental health symptomology (Bassilios et al., 2017; Goel et al., 2021; Green et al., 2015; Kennair et al., 2011; Klag et al., 2016; Rickwood et al., 2015). It must be noted that Cohen’s d effect sizes were consistently not reported and could not be calculated for these studies due to a lack of data reported for key outcomes. The three pre-post studies also examined psychosocial functioning (Green et al., 2015; Klag et al., 2016; Rickwood et al., 2015), and one pre-post study measured hospital admissions (Goel et al., 2021). Five of the six studies (3/3 pre-post, 2/3 controlled) observed significant improvements in mental health symptoms at follow-up, including a reduction in psychological distress (Goel et al., 2021; Rickwood et al., 2015), and improvement in overall symptomology (Goel et al., 2021; Green et al., 2015; Kennair et al., 2011; Klag et al., 2016). All three pre-post studies observed significant improvements in psychosocial functioning after receiving integrated mental health support, including enhanced social, occupational, relational, and physical wellbeing (Green et al., 2015; Klag et al., 2016; Rickwood et al., 2015). Finally, Goel et al. (2021) found that 93% of young people who engaged with the Youth Community Assessment and Treatment Team avoided hospital admission.

#### Lifestyle Programs

Five of the six studies (*n* = 3 controlled, *n* = 2 pre-post) that evaluated lifestyle interventions measured mental health symptomology (Curtis et al., 2016; McGuire et al., 2021; Nasstasia et al., 2017, 2019; Parker et al., 2016) and three studies (*n* = 2 controlled, *n* = 1 pre-post) assessed general psychosocial functioning, which encompassed physical health and activity (Curtis et al., 2016; Parker et al., 2016; Pearce et al., 2020). All five studies that assessed symptomology yielded a significant reduction in depressive symptoms, regardless of study design, with large effect sizes (*d* = 0.84–1.52, Curtis et al., 2016; McGuire et al., 2021; Nasstasia et al., 2017, 2019; Parker et al., 2016). Specifically, 75% of Healthy Body Healthy Mind participants no longer met criteria for major depressive disorder following the intervention (Nasstasia et al., 2017). In the follow-up RCT, 62% no longer met criteria for major depressive disorder, in comparison to 10% of controls (Nasstasia et al., 2019). However, lifestyle interventions did not yield any significant changes in any other symptoms, such as anxiety or substance use, between treatment groups (Parker et al., 2016), nor over time (McGuire et al., 2021). For psychosocial functioning, only one of three studies found significant improvements; those who engaged in KBIM experienced a reduction in weight, improved social functioning and engagement in physical activity compared to the control group (*d* = 0.94, Curtis et al., 2016). In contrast, Pearce et al. (2020) observed a significant increase in the proportion of young people classified as overweight or obese following engagement in their pre-post evaluation of the exercise physiology service, attributed to a lack of program engagement. Similarly, in the RCT of the physical actively lifestyle intervention, intervention participants did not experience significantly better psychosocial functioning than control participants (Parker et al., 2016).

## Discussion

In the last decade, there has been significant reform in Australia to provide young people with appropriate, accessible, and youth-friendly mental health care in the community (McGorry & Mei, 2018). However, no synthesis of the local empirical literature surrounding such programs currently exists. Therefore, this review aimed to: (1) describe the types of community-based mental health programs that have been delivered to Australian youth in the past 10 years; and (2) examine their impact in improving young people’s mental health symptomology and psychosocial functioning. A total of 37 studies (*n* = 13 controlled, *n* = 24 pre-post) were identified, which evaluated four different types of community-based youth mental health programs: (1) therapy, (2) case management, (3) integrated ‘one-stop-shop’, and (4) lifestyle interventions.

The majority of the 37 studies evaluated a therapy program for young people with mild to moderate mental health concerns, which was delivered by a psychologist and typically underpinned by CBT principles and strategies. Most of the therapeutic programs yielded significant improvements in mental health symptoms, with decreased severity and presence of general psychological distress, depressive, anxiety, OCD and substance use symptomology observed over time (Farell et al., 2012; Hudson et al., 2015). This aligns with the extensive international evidence that CBT is a gold standard form of psychotherapy treatment to address these symptoms among young people (David et al., 2018) and supports the application of CBT-based programs with Australian youth. The effectiveness of therapy programs may also be due to the tendency to include parents in the work (i.e., encouraging parents to attend group sessions or targeted sessions for parents to reinforce skill development and strategies at home, Havighurst et al., 2015). Research supports parent participation in youth mental health treatment to facilitate attendance, engagement, and symptom alleviation (Haine-Schlagel & Walsh, 2015). These findings demonstrate the importance of early psychological intervention for young people with mental health concerns to promote positive coping strategies and skills, which can potentially prevent longer-term impacts of a mental disorder (Malla et al., 2016).

Notwithstanding these positive findings for therapeutic programs, it should be highlighted that when a controlled study design was used to evaluate the therapy programs, they did not yield any significant improvements among intervention participants compared to the comparison group (Hides et al., 2011; Poole et al., 2018; Wagner et al., 2017). One explanation for this could be a result of the control groups often receiving a similar kind of therapeutic treatment to that of the intervention group (i.e., standard care that included psychotherapy, modified CBT), which in turn would have limited the treatment effect (Karlsson & Bergmark, 2014). Given this, it is difficult to ascertain whether therapy programs for youth yield significant improvements above and beyond that of standard care; future controlled trials of such programs (i.e., compared to alternative care) are needed as the existing supporting evidence relies heavily on pre-post evaluations (Schley et al., 2018). Further, the large range in effect sizes across the therapy-based studies (i.e., small to large improvements in mental health symptoms) may reduce confidence in their findings. This large variability is likely due to differences in the types of psychotherapy delivered, sample sizes, implementation (i.e., frequency and duration of sessions, group verse individual sessions, etc.), and/or participant engagement across the various programs. However, due to the limited and inconsistent reporting of intervention fidelity and adherence, there remains uncertainty around the full impact of these factors. Therefore, greater transparency in the reporting of intervention activities, implementation, and engagement rates of community-based mental health programs is necessary. An additional drawback of the studies evaluating therapeutic interventions was the lack of examination of psychosocial outcomes (i.e., engagement in education, social relationships, etc.). While reduction in mental health symptomology can contribute to improved broader functioning (Fuhr et al., 2014), this was rarely measured in the reviewed studies. For example, half of the therapy programs were delivered in a group format, which may have provided opportunities to create positive social connections, promote social skills, and foster positive prosocial development, as identified in international reviews (Garcia-Poole et al., 2019; Gardner et al., 2019), yet such outcomes were not assessed. As psychosocial functioning was rarely captured in the reviewed therapeutic studies (both in terms of the focus of the therapeutic intervention and as an evaluated outcome), it remains unclear whether psychotherapy alone can significantly improve young people’s psychosocial functioning.

Consistent with the international literature, both case management and integrated programs were shown to be associated with improvements in mental health symptomology and psychosocial functioning among young people with moderate to severe mental health concerns, with consistently large effect sizes (Daubney et al., 2021; Green et al., 2015). While they differed in mode of delivery, both of these types of models of community mental health care provided intensive, flexible, multidisciplinary, systemic (i.e., engaging with the systems within which the young person exists, such as family, school, peers), wrap-around holistic support that was tailored to each young person’s unique psychosocial needs (i.e., vocational support, social connections, independent skills building). These factors, in combination, have been identified as key components of youth mental health support (Hetrick et al., 2017; Settipani et al., 2019). The positive impact of these programs may also be due to their modes of delivery (i.e., assertive outreach or ‘one-stop-shop’ support), which sought to break down barriers associated with youth service access and engagement (Daubney et al., 2021). This is consistent with the emerging international research supporting the assertive outreach model of case management (i.e., dedicated key worker delivering intensive and flexible outreach support, providing care coordination across various relevant services and systems) to address young people’s psychosocial goals (Vijverberg et al., 2017; Wilson et al., 2017). This assertive outreach model of community mental health care is particularly relevant for youth with severe and complex mental health presentations, as it is flexible, easily accessible, and actively engages and approaches young people in their own environment (Mantzouranis et al., 2019; Vijverberg et al., 2017). In contrast, integrated and center-based “hub-like” programs similarly address key barriers as support is delivered all at the one location (“one-stop-shop”), which provides streamlined support so that young people do not have to navigate a complex mental health system and referral pathways, yet it relies on their attendance at the center (Hetrick et al., 2017; Woody et al., 2019). Indeed, the current findings regarding integrated youth mental health services are consistent with a recent international review of one-stop-shop models of care, which also yielded significant improvements in young people’s psychological distress and psychosocial functioning (Settipani et al., 2019).

The majority of the evidence supporting the case management and integrated programs was based on pre-post evaluations. The reliance on uncontrolled studies evaluating community-based mental health programs for youth has also been reported in international reviews (Farahmand et al., 2012; Garcia-Poole et al., 2019; Vijverberg et al., 2017). Due to the uncontrolled nature of these studies, it is difficult to determine whether the observed improvements in mental health symptomology and psychosocial functioning can be solely attributed to the type of intervention, or due to other factors, such as time or characteristics of clients and treatment settings, which often cannot be distinguished from the effects of the intervention (Cuijpers et al., 2017). Further, given the relatively similar effectiveness of both case management and integrated programs, and the lack of reporting of engagement/attrition rates and effect sizes (particularly for the integrated programs), it is difficult to specifically discern which mode of delivery is more effective, with future evaluation required. Finally, case management was the only type of program that evaluated hospitalizations, demonstrating its potential to reduce the burden on and need for acute mental health services among youth (Conrad et al., 2017). Given that this is one of the key goals of community mental health care reform (McGorry et al., 2018), future research should ensure that hospital admissions are more routinely assessed as a key outcome for such programs to ensure treatment sustainability.

Lifestyle interventions, which focused on exercise and physical activity, were found to consistently alleviate depressive symptoms, regardless of study design (McGuire et al., 2021; Nasstassia et al., 2017, 2019). This aligns with international studies suggesting exercise as a form of behavioral activation to address depressive symptoms (Wegner et al., 2020). However, they did not yield improvements in any other symptoms or psychosocial functioning, with increases in weight and sedentary activity observed (Parker et al., 2016; Pearce et al., 2020). The authors attributed such findings to a lack of engagement. International exercise interventions for youth with mental health concerns have shown strong engagement with components of self-monitoring, positive reinforcement, or rewards (Pascoe et al., 2020). Such adaptations for Australian programs may need to be considered to improve attrition. Further, while the relationship between unhealthy dietary patterns and poor mental health has been demonstrated in children and adolescents (O’Neil et al., 2014), nutrition remains a neglected area of community-based mental health intervention with Australian youth. A notable example of a holistic lifestyle intervention comes from HEALing Matters—a trauma-informed, attachment-focused program that seeks to improve the healthy lifestyle behaviors of young people in out-of-home care (Pizzirani et al., 2019). Further evaluation of community-based lifestyle programs promoting healthy nutrition and physical activity is required to clarify their application in the Australian youth community mental health sector.

### Limitations

While this review has provided a synthesis of the Australian evidence-base of the types and impact of community youth mental health programs, key limitations of the literature must be acknowledged. First, the majority of the reviewed evidence-base relies heavily on low to medium-level quality pre-post evaluations with short-term follow-up (i.e., immediately post-program). The lack of controlled studies, particularly for the case management and integrated programs, makes it difficult to ascertain whether improvements in outcomes are due to the intervention itself, as the potential for bias in the reviewed studies is relatively high (Cuijpers et al., 2017). Youth who meet criteria for such interventions are likely at high risk, experiencing severe or comorbid mental health concerns and complex psychosocial needs, requiring intensive support (Conrad et al., 2017). Due to the ethical issues in randomizing such young people to an organic control group wherein no care is delivered, greater consideration needs to be applied to the delivery of comparative or alternative support. Further, it is recommended that future evaluation studies seek to implement longer-term follow-up so that potential conclusions can be made about the ongoing sustainability of community-based mental health programs on young people’s mental health symptomology and psychosocial functioning into adulthood. Second, there was a lack of community mental health programs that were co-designed or co-created by the young people themselves, or included peer support (i.e., support delivered by those with lived experiences). Only one study specifically described that young people with a lived experience of mental health concerns were involved in the design, development, or delivery of the program (Hall et al., 2021). While peer support has become an integral part of adult community-based mental health service delivery (Shalaby & Agyapong, 2020), this has been largely neglected in the Australian literature for youth thus far. Including the voice of those with lived experiences empowers them as experts, helps to better understand their unique experiences, can overcome barriers to service access, and ensures that supports are specifically tailored to meet their needs (Mulvale et al., 2016; Thabrew et al., 2018). Therefore, future research must prioritize young people’s genuine involvement in the design, development, delivery, and evaluation of mental health programs.

Third, only one reviewed study specifically adopted a trauma-informed lens to support youth with mental health concerns (Klag et al., 2016). Given the high rates of trauma exposure among those with moderate to severe mental health concerns, particularly for those involved in juvenile justice and child welfare systems (Kisiel et al., 2017), it is vital that community mental health services are well-equipped to deliver trauma-informed support. Further, the lack of representation of Aboriginal and Torres Strait Islander youth, other culturally and linguistically diverse (CALD) youth, and LGBTQIA + young people, is a significant gap of the reviewed literature. Given the disproportionate rates of mental health concerns experienced by these populations (AIHW, 2020), there is a greater need for competency, sensitivity, acknowledgment and inclusion of cultural, ethnic, sexuality, and gender diversity among community-based mental health programs for Australian youth. Finally, there was a consistent lack of implementation evaluation and reporting across the included studies. This represents a significant limitation of the literature and compromises the quality of evidence presented as it is unknown whether such programs were delivered as intended. Similarly, numerous studies did not report the level of engagement or attrition throughout program duration, while many noted low engagement. This is consistent with previous international reviews that have also identified poor or a lack of reporting on implementation quality of such programs (Garcia-Poole et al., 2019). Variations in implementation may have also contributed to the large ranges in effect sizes identified across the therapy-based studies, which has similarly been observed in international reviews (Vijverberg et al., 2017). It is well established that when youth do not receive an intervention as intended, it can reduce the effectiveness of the program (Rojas-Andrade & Bahamondes, 2018). Evaluating implementation and uptake is also necessary when establishing or redeveloping mental health programs to avoid replication failure and ensure they effectively engage this population (Ervin et al., 2014). It is necessary that future research focuses on assessing the implementation of youth mental health programs to ensure they are effectively engaging and meeting young people’s needs.

### Implications

The findings of this review highlight the importance of providing youth-friendly, systemic, and integrated assertive outreach support to young people to improve both psychiatric symptoms and psychosocial functioning. Integrated assertive outreach models of community mental health care may overcome barriers to youth accessing support and facilitate the provision of individualized services to improve overall wellbeing. The findings also suggest that community mental health care should be tailored to the unique developmental needs of the young person, which aligns with the recent Inquiry into Mental Health recommendation for a greater focus on person-centered care (Commonwealth of Australia, 2020). Specifically, young people with more severe and complex mental health presentations may require more intensive and holistic support to address their psychosocial needs than therapy alone (McGorry et al., 2018). These findings may also be used to adapt, design, and inform international models of community mental health care for youth. Indeed, the evidence-base provides a useful framework within which both local and international clinicians, health professionals, researchers and policy makers can be guided. The findings may also inform the (re)development and implementation of community mental health programs for youth, with greater focus on the provision of psychosocial support, as outlined in the Inquiry into Mental Health (Commonwealth of Australia, 2020), and particular attention needs to be directed at better supporting the mental health needs of our most vulnerable youth (i.e., out-of-home care, ATSI, CALD, LGBTQIA + etc.). Finally, in recognizing that the majority of reviewed studies provided support to young people aged 12–25, it is recommended that the current transition from youth to adult community mental health services is improved to overcome the widely documented barriers associated with the discontinuation of support at 18 and to ensure continuity of mental health care (Embrett et al., 2016; Nguyen et al., 2017). Ideally, the aim of youth services should be to reduce the need for transition into adult services (McGorry et al., 2018). Therefore, it is necessary to strengthen integrated models of community mental health care for Australian youth across this broader age range. A summary of key recommendations to address existing limitations of the current evidence-base and guide future research and practice includes the following:

- Higher quality program evaluations (including appropriate control groups) with longer-term follow-up
- Greater transparency in the reporting of intervention activities, implementation, fidelity, and effect sizes
- Greater focus on the provision of psychosocial support and evaluation of psychosocial-related outcomes as part of community-based mental health programs
- Implementation and evaluation of community-based mental health programs that appropriately support diverse youth, including youth from out-of-home care, intergenerational disadvantage or low socioeconomic status, and youth that identify as ATSI, CALD, or LGBTQIA + 
- Implementation and evaluation of trauma-informed approaches to community-based mental health support
- Prioritize young people’s genuine involvement in the design, development, delivery, and evaluation of mental health programs, including co-design and participatory approaches

The authors acknowledge that while some of this work may already be occurring in practice in the local context, it is underrepresented in the empirical literature. Further research across these areas may contribute to a more comprehensive and transparent evidence-base to inform the development and implementation of community mental health programs in Australia that yield sustained improvements in young people’s wellbeing.

## Conclusion

This review has described and examined the types and impact of Australian community mental health programs for youth. The current findings suggest that an integrated assertive outreach model of youth mental health care may facilitate the provision of therapeutic support, other individualized services (i.e., vocational support, social development, drug and alcohol, independent skills building), and systemic support (i.e., family, school, peers) to young people experiencing significant mental health concerns. However, higher-quality research is needed to more confidently conclude which type of community-based mental health intervention can yield sustainable improvements in young people’s outcomes. Recommendations for future research and implications for practice have been provided. Overall, the literature suggests that greater focus on psychosocial functioning, both in terms of program focus and program outcomes, is needed, as well as mental health support that is trauma-informed and co-designed, and greater transparency in evaluation reporting. Advancing the evidence-base of community mental health programs is highly warranted to improve the life trajectory of Australian youth.

## Supplementary Information

Below is the link to the electronic supplementary material.

Supplementary file1 (DOCX 178 KB)

## Data Availability

Data sharing is not applicable to this work as it is a systematic literature review, so no datasets were generated or analyzed.

## Footnotes

1 Refers to a person’s sexual orientation or gender identity, including lesbian, gay, bisexual, transgender or gender diverse, queer, intersex, or asexual.

2 The age range *and* mean age of study participants encompassed 10–25 years inclusive.

3 Australia was not included as a search term so that studies that did not specify ‘Australia’ as the study location (i.e., reference to specific city, town, state, or territory only) were not missed.

4 Five of the 37 studies did not report the average age of their sample; only the age range was provided.

5 The provision of in-house or “hub-like” multidisciplinary clinical and psychosocial support all available at the one location.

## References

[CR1] Adriaanse M, Veling W, Doreleijers T, van Domburgh L (2014). The link between ethnicity, social disadvantage and mental health problems in a school-based multiethnic sample of children in the Netherlands. European Child and Adolescent Psychiatry.

[CR2] Agnafors S, Barmark M, Sydsjo G (2020). Mental health and academic performance: A study on selection and causation effects from childhood to early adulthood. Social Psychiatry and Psychiatric Epidemiology.

[CR510] American Psychiatric Association (APA). (2013). *Diagnostic and statistical manual of mental disorders* (5th ed.). APA.

[CR3] Anderson JK, Howarth E, Vainre M, Jones PB, Humphrey A (2017). A scoping literature review of service-level barriers for access and engagement with mental health services for children and young people. Children and Youth Services Review.

[CR4] Australian Bureau of Statistics. (2016). *National Aboriginal and Torres Strait Islander Social Survey, 2014–15*. Retrieved January 28, 2022, from https://www.abs.gov.au/ausstats/abs@.nsf/Lookup/by%20Subject/4714.0~2014-15~Feature%20Article~Aboriginal%20and%20Torres%20Strait%20Islander%20people%20with%20a%20mental%20health%20condition%20(Feature%20Article)~10

[CR5] Australian Institute of Health and Welfare. (2020). *Australia’s children – children with mental illness. Cat. no: CWS 69.* Retrieved January 28, 2022, from https://www.aihw.gov.au/reports/children-youth/australias-children/contents/health/children-mental-illness

[CR6] Australian Institute of Health and Welfare. (2021). *Mental health services in Australia – COVID-19 impact on mental health.* Retrieved January 28, 2022, from https://www.aihw.gov.au/reports/mental-health-services/mental-health-services-in-australia/report-contents/mental-health-impact-of-covid-19

[CR7] *Bassilios, B., Telford, N., Rickwood, D., Spittal, M. J., & Pirkis, J. (2017). Complementary primary mental health programs for young people in Australia: Access to Allied Psychological Services (ATAPS) and Headspace. *International Journal of Mental Health Systems, 11*(19), 1–11. 10.1186/s13033-017-0125-7 10.1186/s13033-017-0125-7 PMC5301357 28203274

[CR9] *Brewer, W., Lambert, T., Witt, K., Dileo, J., Duff, C., Crlenjak, C., McGorry, P. D., & Murphy, B. P. (2015). Intensive case management for high-risk patients with first-episode psychosis: Service model and outcomes. *Lancet Psychiatry, 2*(1), 29–37. 10.1016/S2215-0366(14)00127-8 10.1016/S2215-0366(14)00127-8 26359610

[CR10] Caspi A, Houts R, Ambler A (2020). Longitudinal assessment of mental health disorders and comorbidities across 4 decades among participants in the Dunedin birth cohort study. JAMA.

[CR11] *Chia, A., Assan, B., Finch, E., Stargatt, R., Burchell, P., Jones, H., Heywood-Smith, J. (2013). Innovations in practice: Effectiveness of specialist adolescent outreach service for at-risk adolescents. *Child and Adolescent Mental Health, 18*(2), 116–119. 10.1111/j.1475-3588.2012.00654.x 10.1111/j.1475-3588.2012.00654.x 32847289

[CR12] Cohen J (1988). Statistical power analysis for the behavioural sciences.

[CR13] Commonwealth of Australia. (2020). *Mental health productivity commission inquiry report.* Retrieved January 28, 2022, from https://www.pc.gov.au/inquiries/completed/mental-health/report/mental-health-volume1.pdf

[CR14] *Conrad, A., Lewin, T., Sly, K., Schall, U., Hunter, M., & Carr, V. J. (2017). Utility of risk-status for predicting psychosis and related outcomes: evaluation of a 10-year cohort of presenters to a specialised early psychosis community mental health service. *Psychiatry Research, 247,* 336–344. 10.1016/j.psychres.2016.12.005 10.1016/j.psychres.2016.12.005 27984822

[CR15] Costello EJ, He JP, Sampson NA, Kessler RC, Merikangas KR (2014). Services for adolescents with psychiatric disorders: 12-month data from the National Comorbidity Survey-Adolescent. Psychiatric Services.

[CR16] Cuijpers P, Weitz E, Cristea I, Twisk J (2017). Pre-post effect sizes should be avoided in meta-analyses. Epidemiology and Psychiatric Sciences.

[CR17] *Curtis, J., Watkins, A., Rosenbaum, S., Teasdale, S., Kalucy, M., Samaras, K., & Ward, P. B. (2016). Evaluating an individualised lifestyle and life skills intervention to prevent antipsychotic-induced weight gain in first-episode psychosis. *Early Intervention in Psychiatry, 10*(3), 267–276. 10.1111/eip.12230 25721464

[CR18] Das J, Salam R, Lassi Z, Khan M, Mahmood W, Patel V, Bhutta Z (2016). Interventions for adolescent mental health: An overview of systematic reviews. Journal of Adolescent Health.

[CR19] *Daubney, M. F., Raeburn, N., Blackman, K., Jeffries, H., & Healy, K. L. (2021). Outcomes of assertive community treatment for adolescents with complex mental health problems who are difficult to engage. *Journal of Child and Family Studies, 30*(1), 502-516. 10.1007/s10826-020-01882-3

[CR20] David D, Cristea I, Hofmann S (2018). Why cognitive behavioural therapy is the current gold standard of psychotherapy. Frontiers Psychiatry.

[CR21] Dray J, Bowman J, Campbell E (2017). Systematic review of universal resilience-focused interventions targeting child and adolescent mental health in the school setting. Journal of the American Academy of Child & Adolescent Psychiatry.

[CR22] *Edwards, C., & Hegerty, S. (2018). Where it’s cool to be kitty: An art therapy group for young people with mental health issues using origami and mindfulness. *Social Work with Groups, 41*(1–2), 151–164. 10.1080/01609513.2016.1258625.

[CR23] Embrett M, Randall G, Longo C, Nguyen T, Mulvale G (2016). Effectiveness of health system services and programs for youth to adult transitions in mental health care: A systematic review of academic literature. Administration and Policy in Mental Health.

[CR24] Ervin K, Phillips J, Tomnay J (2014). Establishing a clinic for young people in a rural setting: A community initiative to meet the needs of rural adolescents. Australian Journal of Primary Health.

[CR25] Evensen M, Lyngstad T, Melkevik O, Reneflot A, Mykletun A (2017). Adolescent mental health and earnings inequalities in adulthood: Evidence from the Young-HUNT study. Journal of Epidemiology and Community Health.

[CR26] Farahmand FK, Duffy SN, Tailor MA (2012). Community-based mental health and behavioural programs for low-income urban youth: A meta-analysis review. Clinical Psychology: Science and Practice.

[CR27] *Farrell, L., Waters, A., Milliner, A., & Ollendick, T. (2012). Comorbidity and treatment response in paediatric obsessive-compulsive disorder: A pilot study of group cognitive-behavioural treatment. *Psychiatry Research, 199*(2), 115–123. 10.1016/j.psychres.2012.04.035 10.1016/j.psychres.2012.04.035 22633155

[CR28] Fuhr DC, Salisbury TT, De Silva MJ, Atif N, van Ginneken N, Rahman A, Patel V (2014). Effectiveness of peer-delivered interventions for severe mental illness and depression on clinical and psychosocial outcomes: A systematic review and meta-analysis. Social Psychiatry and Psychiatric Epidemiology.

[CR29] Garcia-Poole C, Sonia B, Jose RM (2019). How do communities intervene with adolescents at psychosocial risk? A systematic review of positive development programs. Children and Youth Services Review.

[CR30] Gardner A, Filia K, Killackey E, Cotton S (2019). The social inclusion of young people with serious mental illness: A narrative review of the literature and suggested future directions. Australian and New Zealand Journal of Psychiatry.

[CR31] *Goel, C., Shafi, R., Conner, A. J., Waters, F., Croarkin, P. E., & McGorry, P. (2021). A retrospective evaluation of a pilot youth community assessment and treatment service. *Psychiatric Services, 72*(4), 415–420. 10.1176/appi.ps.201900013 10.1176/appi.ps.201900013 33557596

[CR32] *Green, J., Melvin, G. A., Newman, L., Jones, M., Taffe, J., & Gordon, M. (2015). Day program for young people with anorexia nervosa. *Australasian Psychiatry,* 1–5. 10.1177/1039856215584513 10.1177/1039856215584513 25948510

[CR33] Haine-Schlagel R, Walsh N (2015). A review of parent participation engagement in child and family mental health treatment. Clinical Child and Family Psychology Review.

[CR34] *Hall, K., Youssef, G., Simpson, A., Sloan, E., Graeme, L., Perry, N., Moulding, R., Baker, A. L., Beck, A. K., & Staiger, P. K. (2021). An emotion regulation and impulse control (ERIC) intervention for vulnerable young people: A multi-sectoral pilot study. *Frontiers in Psychology, 12,* 1–12. 10.3389/fpsyg.2021.554100 10.3389/fpsyg.2021.554100 PMC8047628 33868064

[CR35] Hall S, Fildes J, Perrens B, Plummer J, Carlisle E, Cockayne N, Werner-Seidler A (2019). Can we talk? Seven year youth mental health report - 2012–2018.

[CR36] *Havighurst, S. S., Kehoe, C. E., & Harley, A. E. (2015). Tuning in to teens: Improved parental responses to anger and reducing youth externalising behaviour problems*. Journal of Adolescence, 42,* 148–158. 10.1016/j.adolescence.2015.04.005 10.1016/j.adolescence.2015.04.005 26005933

[CR37] *Hayes, L., Boyd, C., & Sewell, J. (2011). Acceptance and commitment therapy for the treatment of adolescent depression: A pilot study in a psychiatric outpatient setting. *Mindfulness, 86*(2), 86–94.

[CR38] Hetrick S, Bailey A, Smith K, Malla A, Mathias S, Singh S, O’Reilly A, Verma S, Benoit L, Fleming T, Moro M, Rickwood D, Duffy J, Eriksen T, Illback R, Fisher CA, McGorry PD (2017). Integrated (one-stop shop) youth health care: Best available evidence and future directions. Medical Journal of Australia.

[CR39] *Hickey, T., Nelson, B., Enticott, J., & Meadows, G. (2020). The MAC-P program: A pilot study of a mindfulness and compassion program for youth with psychotic experiences. *Early Intervention in Psychiatry, 15*(5), 1326–1334. 10.1111/eip.13085 10.1111/eip.13085 33340259

[CR40] *Hides, L., Elkins, K., Scaffidi, A., Cotton, S. M., Carroll, S., & Lubman, D. (2011). Does the addition of integrated cognitive behaviour therapy and motivational interviewing improve the outcomes of standard care for young people with comorbid depression and substance use. *Medical Journal of Australia, 195*(3), S31–S37. 10.5694/j.1326-5377.2011.tb03263.x 10.5694/j.1326-5377.2011.tb03263.x 21806516

[CR41] Hollis C, Falconer C, Martin J, Whittington C, Stockton S, Glazebrook C, Davies E (2016). Annual research review: Digital health interventions for children and young people with mental health problems – a systematic and meta-review. The Journal of Child Psychology and Psychiatry.

[CR42] *Hopkins, L., Lee, S., McGrane, T., & Barbara-May, R. (2017). Single session family therapy in youth mental health: can it help? *Australasian Psychiatry, 25*(2), 108–111. 10.1177/1039856216658807 10.1177/1039856216658807 27469418

[CR43] *Howe, D., Batchelor, S., & Coates, D. (2017). Young Australians with moderate to severe mental health problems: Client data and outcomes at children and young people’s mental health. *Early Intervention in Psychiatry, 11*(4), 334–341. 10.1111/eip.12252 10.1111/eip.12252 25962783

[CR44] *Hudson, J., Rapee, R., Lyneham, H., McLellan, L. F., Wuthrich, V. M., & Schniering, C.A. (2015). Comparing outcomes for children with different anxiety disorders following cognitive behavioural therapy. *Behaviour Research and Therapy, 72,* 30–37. 10.1016/j.brat.2015.06.007 10.1016/j.brat.2015.06.007 26164621

[CR45] Hughes F, Hebel L, Badcock P, Parker AG (2016). Ten guiding principles for youth mental health services. Early Intervention in Psychiatry.

[CR46] Karlsson P, Bergmark A (2014). Compared with what? An analysis of control-group types in Cochrane and Campbell reviews of psychosocial treatment efficacy with substance use disorders. Addiction.

[CR47] *Kehoe, C. E., Havighurst, S. S., & Harley, A. E. (2014). Tuning in to teens: Improving parent emotion socialisation to reduce youth internalising difficulties. *Social Development, 23*(2), 413–431. 10.1111/sode.12060

[CR48] *Kennair, N., Mellor, D., & Brann, P. (2011). Evaluating the outcomes of adolescent day programs in an Australian child and adolescent mental health service. *Clinical Child Psychology and Psychiatry, 16*(1), 21–31. 10.1177/1359104509340951 10.1177/1359104509340951 20404071

[CR49] *Killackey, E., Allott, K., Jackson, H. J., Scutella, R., Tseng, Y., Borland, J., Proffitt, T., Hunt, S., Kay-Lambkin, F., Chinnery, G., Baksheev, G., Alvaraz-Jimenez, M., McGorry, P. D., & Cotton, S. M. (2019). Individual placement and support for vocational recovery in first episode psychosis: Randomised controlled trial. *The British Journal of Psychiatry, 214*(2), 76–82. 10.1192/bjp.2018.191 10.1192/bjp.2018.191 30251616

[CR50] *Killackey, E., Allott, K., Woodhead, G., Connor, S., Dragon, S., & Ring, J. (2017). Individual placement and support, supported education in young people with mental illness: An exploratory feasibility study. *Early Intervention in Psychiatry, 11*(6), 526–531. 10.1111/eip.12344 10.1111/eip.12344 27121481

[CR51] Kisiel C, Summersett-Ringgold F, Weil L, McCelland G (2017). Understanding strengths in relation to complex trauma and mental health symptoms within child welfare. Journal of Child and Family Studies.

[CR52] *Klag, S., Fox, T., Martin, G., Eadie, K., Bergh, W., Keegan, F., Turner, D., & Raeburn, N. (2016). Evolve therapeutic services: A 5-year outcome study of children and young people in out-of-home care with complex and extreme behavioural and mental health problems. *Children and Youth Services Review, 69,* 268–274. 10.1016/j.childyouth.2016.08.017

[CR53] Kwok K, Yuan S, Ougrin D (2016). Review: Alternatives to inpatient care for children and adolescents with mental health disorders. Child and Adolescent Mental Health.

[CR54] LGBTIQ+ Health Australia. (2021). *Snapshot of mental health and suicide prev**ention statistics for LGBTIQ+ people.* Retrieved January 28, 2022, from https://d3n8a8pro7vhmx.cloudfront.net/lgbtihealth/pages/549/attachments/original/1620871703/2021_Snapshot_of_Mental_Health2.pdf?1620871703

[CR55] Malla A, Iyer S, McGorry P, Cannon M, Coughlan H, Singh S, Jones P, Joober R (2016). From early intervention in psychosis to youth mental health reform: A review of the evolution and transformation of mental health services for young people. Social Psychiatry and Psychiatric Epidemiology.

[CR56] Mantzouranis G, Baier V, Holzer L, Urben S, Villard E (2019). Clinical significance of assertive community treatment among adolescents. Social Psychiatry and Psychiatric Epidemiology.

[CR57] McGorry P, Bates T, Birchwood M (2018). Designing youth mental health services for the 21^st^ century: Examples from Australia, Ireland, and the UK. The British Journal of Psychiatry.

[CR58] McGorry P, Mei C (2018). Early intervention in youth mental health: Progress and future directions. Evidence Based Mental Health.

[CR59] *McGuire, D., Shannon, A., Somaiya, J., Brown, E., & O’Donoghue, B. (2021). A pilot study of a yoga intervention for the treatment of anxiety in young people with early psychosis. *Early Intervention in Psychiatry,* 1–5. 10.1136/ebmental-2018-300060 10.1111/eip.13151 33929083

[CR60] Mulvale A, Miatello A, Hackett C, Mulvale G (2016). Applying experience-based co-design with vulnerable populations: Lessons from a systematic review of methods to involve patients, families and service providers in child and youth mental health service improvement. Patient Experience Journal.

[CR61] *Nasstasia, Y., Baker, A. L., Halpin, S. A., Lewin, T. J., Hides, L., Kelly, B. J., & Callister, R. (2017). Pilot study of an exercise intervention for depressive symptoms and associated cognitive behavioural factors in young adults with major depression. The *Journal of Nervous and Mental Disease, 205*(8), 647–655. 10.1097/NMD.0000000000000611 10.1097/NMD.0000000000000611 27805982

[CR62] *Nasstasia, Y., Baker, A., Lewin, T., Halpin, S. A., Hides, L., Kelly, B. J., & Callister, R. (2019). Differential treatment effects of an integrated motivational interviewing and exercise intervention on depressive symptom profiles and associated factors: A randomised controlled cross-over trial among youth with major depression. *Journal of Affective Disorders, 259*(5), 413–423. 10.1016/j.jad.2019.08.035 10.1016/j.jad.2019.08.035 31610998

[CR500] National Institute of Health. (2021). *Study quality assessment tools*. Retrieved February 9, 2022, from https://www.nhlbi.nih.gov/healthtopics/study-quality-assessment-tools

[CR63] Nguyen T, Embrett MG, Barr NG, Mulvale GM, Vania DK, Randall GE, DiRezze B (2017). Preventing youth from falling through the cracks between child/adolescent and adult mental health services: A systematic review of models of care. Community Mental Health Journal.

[CR64] O’Keeffe L, O’Reilly A, O’Brien G, Buckley R, Illback R (2015). Description and outcome evaluation of Jigsaw: An emergent Irish mental health early intervention programme for young people. Irish Journal of Psychological Medicine.

[CR65] O’Neil A, Quirk SE, Housden S, Brennan SL, Williams LJ, Pasco JA, Berk M, Jacka FN (2014). Relationship between diet and mental health in children and adolescents: A systematic review. American Journal of Public Health.

[CR66] Organisation for Economic Co-operation and Development [OECD]. (2019). *Supporting young people’s mental health through the COVID-19 crisis*. In Tackling Coronavirus: Contributing to a global effort. OECD.

[CR67] Page M, McKenzie J, Bossuyt P (2021). The PRISMA 2020 statement: An updated guideline for reporting systematic reviews. BMJ.

[CR68] *Parker, A., Hetrick, S., Jorm, A., Mackinnon, A. J., McGorry, P. D., Yung, A. R., Scanlan, F., Stephens, J., Baird, S., Moller, B., & Purcell, R. (2016). The effectiveness of simple psychological and physical activity interventions for high prevalence mental health problems in young people: A factorial randomised controlled trial. *Journal of Affective Disorders, 196,* 200–209. 10.1016/j.jad.2016.02.043 10.1016/j.jad.2016.02.043 26926659

[CR69] Pascoe M, Bailey AP, Craike M, Carter T, Patten R, Stepto N, Parker A (2020). Physical activity and exercise in youth mental health promotion: A scoping review. BMJ Open Sport and Exercise Medicine.

[CR70] *Pearce, M., Foote, L., Brown, E., & O’Donoghue, B. (2020). Evaluation of an exercise physiology service in a youth mental health service. *Irish Journal of Psychological Medicine, 19,* 1–6. 10.1017/ipm.2020.91 10.1017/ipm.2020.91 32811583

[CR71] Pizzirani B, O’Donnell R, Bruce L, Breman R, Smales M, Xie J, Hu H, Skouteris H, Green R (2019). The large-scale implementation and evaluation of a healthy lifestyle program in residential out-of-home care: Study protocol. International Journal of Adolescence and Youth.

[CR72] *Poole, L. A., Knight, T., Toumboutou, J. W., Lubman, D. I., Bertino, M. D., & Lewis, A. J. (2018). A randomised controlled trial of the impact of a family-based adolescent depression intervention on both youth and parent mental health outcomes. *Journal of Abnormal Child Psychology, 46*(1), 169–181. 10.1007/s10802-017-0292-7 10.1007/s10802-017-0292-7 28374218

[CR73] *Porter, M., & Nuntavisit, L. (2016). An evaluation of multisystemic therapy with Australian families. *Australian and New Zealand Journal of Family Therapy, 37*(4), 443–462. 10.1002/anzf.1182 10.1002/anzf.1182 PMC5599972 28979064

[CR74] Purcell R, Harrigan S (2017). Lifetime rates and correlates of crime victimisation in young people with mental ill health. Australasian Psychiatry.

[CR75] Radez J, Reardon T, Creswell C, Lawrence P, Evdoka-Burton G, Waite P (2021). Why do children and adolescents (not) seek and access professional help for their mental health problems? A systematic review of quantitative and qualitative studies. European Child and Adolescent Psychiatry.

[CR76] *Rickwood, D. J., Mazzer, K. R., Telford, N. R., Parker, A. G., Tanti, C. J., & McGorry, P. D. (2015). Changes in psychological distress and psychosocial functioning in young people accessing Headspace centres for mental health problems. *Medical Journal of Australia, 202*(10), 537–543. 10.5694/mja14.01696 10.5694/mja14.01696 26021366

[CR77] Rickwood D, Paraskakis M, Quin D, Hobbs N, Ryall V, Trethowan J, McGorry P (2019). Australia’s innovation in youth mental health care: The Headspace centre model. Early Intervention in Psychiatry.

[CR78] Rocha T, Graeff-Martins A, Kieling C, Rohde L (2015). Provision of mental healthcare for children and adolescents: A worldwide view. Current Opinion in Psychiatry.

[CR79] Rojas-Andrade R, Bahamondes L (2018). Is implementation fidelity important? A systematic review on school-based mental health programs. Contemporary School Psychology.

[CR80] Rosen A, Gurr R, Fanning P (2010). The future of community-centred health services in Australia: Lessons from the mental health sector. Australian Health Review.

[CR81] *Sabbioni, D., Feehan, S., Nicholls, C., Soong, W., Rigoli, D., Follett, D., Carastathis, G., Gomes, A., Griffiths, J., Curtis, K., Smith, W., & Waters, F. (2018). Providing culturally informed mental health services to Aboriginal youth: The YouthLink model in Western Australia. *Early Intervention in Psychiatry, 12*(5), 987–994. 10.1111/eip.12563 10.1111/eip.12563 29573565

[CR82] Sapiro B, Ward W (2020). Marginalised youth, mental health, and connection with others: A review of the literature. Child and Adolescent Social Work Journal.

[CR83] *Schley, C., Pace, N., Mann, R., McKenzie, C., McRoberts, A., & Parker, A. (2018). The Headspace brief interventions clinic: Increasingly timely access to effective treatments for young people with early signs of mental health problems. *Early Intervention in Psychiatry, 13*(5), 1073–1082. 10.1111/eip.12729 10.1111/eip.12729 30160372

[CR84] *Schley, C., Yuen, K., Fletcher, K., & Radovini, A. (2012). Does engagement with an intensive outreach service predict better treatment outcomes in ‘high-risk’ youth? *Early Intervention in Psychiatry, 6*(2), 176–184. 10.1111/j.1751-7893.2011.00338.x 10.1111/j.1751-7893.2011.00338.x 22273358

[CR85] Settipani C, Hawke LD, Cleverley K, Chaim G, Cheung A, Mehra K, Rice M, Szatmari P, Henderson J (2019). Key attributes of integrated community-based youth service hubs for mental health: A scoping review. International Journal of Mental Health Systems.

[CR86] Shalaby RAH, Agyapong VIO (2020). Peer support in mental health: Literature review. JMIR Mental Health.

[CR87] *Tan, L., & Martin, G. (2015). Taming the adolescent mind: a randomised controlled trial examining clinical efficacy of an adolescent mindfulness-based group program. *Child and Adolescent Mental Health, 20*(1), 49–55. 10.1111/camh.12057 10.1111/camh.12057 32680328

[CR88] Tarren-Sweeney M (2018). The mental health of adolescents residing in court-ordered foster care: Findings from a population survey. Child Psychiatry & Human Development.

[CR89] Thabrew H, Fleming T, Hetrick S, Merry S (2018). Co-design of eHealth interventions with children and young people. Frontiers in Psychiatry.

[CR90] UNICEF. (2021). The state of the world’s children 2021. *On my mind: promoting, protecting and caring for children’s mental health*. UNICEF.

[CR92] Velasco AA, Cruz ISS, Billings J, Jimenez M, Rowe S (2020). What are the barriers, facilitators and interventions targeting help-seeking behaviours for common mental health problems in adolescents? A systematic review. BMC Psychiatry.

[CR93] Vijverberg R, Ferdinand R, Beekman A, van Meijel B (2017). The effect of youth assertive community treatment: A systematic PRISMA review. BMC Psychiatry.

[CR94] Vyas NS, Birchwood M, Singh SP (2015). Youth services: Meeting the mental health needs of adolescents. Irish Journal of Psychological Medicine.

[CR95] *Wagner, G. A., Mildred, H., Gee, D., Black, E. B., & Brann, P. (2017). Effectiveness of brief intervention and case management for children and adolescents with mental health difficulties. *Children and Youth Services Review, 79,* 362–367. 10.1016/j.childyouth.2017.06.046

[CR96] Wegner M, Amatriain-Fernandez S, Kaulitzky A (2020). Systematic review of meta-analyses: Exercise effects on depression in children and adolescents. Frontiers and Psychiatry.

[CR97] *Westwater, J. J., Murphy, M., Handley, C., & McGregor, L. (2020). A mixed methods exploration of single session family therapy in a child and adolescent mental health service in Tasmania, Australia. *Australian and New Zealand Journal of Family Therapy, 41*(3), 258–270. 10.1002/anzf.1420

[CR98] Wilson J, Clarke T, Lower R (2017). Creating an innovative youth mental health service in the United Kingdom: The Norfolk Youth Service. Early Intervention in Psychiatry.

[CR99] Woody C, Baxter A, Wright E (2019). Review of services to inform clinical frameworks for adolescents and young adults with severe, persistent and complex mental illness. Clinical Child Psychology and Psychiatry.

[CR100] World Health Organisation. (2020). *Adolescent mental health*. Retrieved January 28, 2022, from https://www.who.int/news-room/fact-sheets/detail/adolescent-mental-health

