# Supplementary material for: The Impact of Community Mental Health Programs for Australian Youth: A Systematic Review
Source: Clin Child Fam Psychol Rev. 2022 Feb 16;25(3):573–90. doi: 10.1007/s10567-022-00384-6 (PMC8853061; doi:10.1007/s10567-022-00384-6)
Supplement: Supplementary file 1 — Supplementary file1 (DOCX 178 KB) [file 10567_2022_384_MOESM1_ESM.docx]

**The Impact of Community Mental Health Programs for Australian Youth: A Systematic Review**

**Supplementary Tables**

Melissa Savaglio^1^, Renee O’Donnell^1^, Kostas Hatzikiriakidis^1^, Dave Vicary^2^, Helen Skouteris^1, 3^

^1^Health and Social Care Unit, School of Public Health and Preventive Medicine, Monash University, Melbourne, Australia

^2^Baptcare, Melbourne, Australia

^3^Warwick Business School, University of Warwick, Coventry, United Kingdom

***Corresponding Author:** melissa.savaglio@monash.edu

Supplementary Table 1. Search Terms___________________________________________2

Supplementary Table 2. Summary Studies*______________________________________*__3

Supplementary Table 3. Summary of Results_____________________________________18

Supplementary Table 4. Quality Assessment_____________________________________30

Supplementary Table 1. *Search Terms*

| **Concept** | **Search terms** |
| --- | --- |
| Youth | Child* OR prepubescen* OR pubescen* OR preteen* OR teen* OR puberty OR adolescen* OR youth OR young* people OR young* person* OR young* adult* OR juvenile* |
| Mental health | Mental illness* OR mental disorder* OR mental health OR mental ill health OR mental health symptom* OR psych* symptom* OR psychological disorder* OR psychosis OR psychotic OR schizo* OR depress* OR anxiety OR anxious OR mood disorder* OR bipolar OR conduct disorder* OR oppositional defiant disorder* OR substance abuse OR substance use OR substance dependence OR eating disorder* OR posttraumatic stress disorder OR PTSD OR obsessive compulsive disorder OR OCD OR personality disorder* OR suicid* |
| Community-based program | Community adj4 program* OR community adj4 intervention* OR community adj4 treatment* OR community adj4 support* OR community adj4 care OR community adj4 service* OR community adj4 therap*” OR community mental health OR outreach OR home visit* |

Supplementary Table 2. *Summary of Study, Participant, and Intervention Characteristics*

| **Therapy Programs** | | | | | | | |
| --- | --- | --- | --- | --- | --- | --- | --- |
| **First Author (Year), State** | **Study Design** | | **Participant**  **Characteristics** | **Intervention**  **Description** | | **Comparison Description** | **Duration and Frequency** |
| Edwards (2018), Queensland | Pre-post | | Sample Size: *n*=4  Age Range: 14-16 years  Mean Age (SD): NR  Gender (Female): 100%  Mental health concerns: Any mental health diagnosis (types not specified)  Ethnicity: NR  CALD: NR  LGBTQIA+ identity: NR  Family socioeconomic status: NR | “Goodbye Kitty” Origami and Mindfulness-based Art Therapy Group Program:  Group sessions were conducted in the early evenings at Kooky Clinic, a community mental health clinic that aims to provide a nonclinical, creative, youth-friendly space where it’s ‘cool to be different’. The sessions took place in a customized yurt to create an exotic, fun and unique therapeutic environment, and emphasised social interaction in the group setting. Each session involved a check-in activity (i.e., to allow participants to settle in, using a range of visual and tactile materials to prompt sharing), an origami activity (i.e., participants created their own origami figures, such as hearts, flowers, or animals), and a mindfulness activity (i.e., mindful eating, breathing, or guided meditation).  Community setting: community mental health clinic | | Not applicable | Weekly 1.5-hour sessions, at least four sessions |
| Farrell (2012), Queensland | Pre-post | | Sample Size: *n*=43  Age Range: 7-17 years  Mean Age (SD): 11.09 (2.52)  Gender (Female): 30%  Mental health concerns: 100% obsessive–compulsive disorder, and 35% with comorbid pervasive developmental disorders, 19% comorbid attention deficit/hyperactivity disorder and 12% comorbid depression.  Ethnicity: NR  CALD: NR  LGBTQIA+ identity: NR  Family socioeconomic status: average family income ranged from $61,000-$80,000 | Group Cognitive Behavioural Therapy (CBT) for Obsessive Compulsive Disorder:  A manualised family-based CBT group program was delivered at the University psychology clinic. Treatment involved 13 weekly group sessions and 2 booster sessions (1 month and 3 months post-treatment), with 15-minutes of parental involvement at the end of each session, delivered by post-graduate level clinicians. Parents received three structured group sessions (1 hour), and two individual family review sessions (1 hour, including the child and their parents and siblings if desired) at week 5 and week 10 of the program. The child group sessions focused on psychoeducation, cognitive training, anxiety management training, developing stimulus hierarchies, graded exposure and response prevention (ERP), and relapse prevention. Parent group sessions focused on psychoeducation, problem- solving skills, strategies to reduce parental involvement in the child’s symptoms, and encouraging family support of home-based ERP.  Community setting: University psychology clinic | | Not applicable | 13 weeks duration, 13 weekly 1.5-hour sessions |
| Hall (2021), New South Wales, Queensland, and Victoria | Pre-post | | Sample Size: *n*=79  Age Range: 16-25 years  Mean Age (SD): 19.30 (2.94)  Gender (Female): 47%  Mental health concerns: Depression and anxiety  Ethnicity: 20% Aboriginal and/or Torres Strait Islander  CALD: 8% not born in Australia, location not specified  LGBTQIA+ identity: 2.5% non-binary/gender queer  Family socioeconomic status: NR | Emotion Regulation and Impulse Control (ERIC) Intervention:  An emotion regulation and impulse control modularised CBT skills-based program was delivered by clinicians (e.g., psychologists, counsellors, social workers etc). ERIC organises emotion regulation exercises, skills and processes into eight domains: (1) reducing vulnerability; (2) emotional literacy; (3) flexible thinking; (4) allowing; (5) micro mindfulness; (6) tolerating discomfort; (7) decision making; (8) identity and values. The processes and strategies within each domain were addressed through a series of 15 exercises that were presented in worksheets and tools. Each exercise included the following elements: (a) psychoeducation delivered via a narrative, experiential exercise, or analogy; (b) a behavioural exercise involving node-link mapping and (c) a practice and reflection schedule to prompt daily repetition of new skills.  Community setting: outreach (location not specified), community centres and clinics | | Not applicable | 12 weeks duration, frequency not reported |
| Havighurst (2015), Victoria | Randomised controlled trial | | Sample Size: *n*=224  Age Range: 10-13 years  Mean Age (SD): 12.01 (0.42)  Gender (Female): 51%  Mental health concerns: Youth experiencing externalising mental health concerns  Ethnicity: 90% Caucasian; 8% Asian; 2% African  CALD: NR  LGBTQIA+ identity: NR  Family socioeconomic status: 80% of parents in paid employment; 76% completed high school, 44% completed bachelor’s degree or higher; 39% had gross annual combined family income of $100,000 or more, 32% between $60,000-$99,000 | Tuning into Teens:  Tuning into Teens is a manualised 6-session group parenting program that teaches parents how to respond to their teen's emotions in ways that aim to increase communication and connection while helping the adolescent to understand their emotions. Parents were taught strategies for understanding and managing their own emotions and were educated about adolescent emotional development. The intervention was delivered through didactic teaching, role plays, exercises, home activities, and a DVD. Group sessions were conducted at a local community centre, with 6-13 participants per group. Primary facilitators were psychologists.  Community setting: community centre | | Control:  Parents did not receive Tuning into Teens. No intervention provided to control parents. | 6 weeks duration, one 2-hour session per week. |
| Hayes (2011), Victoria | Quasi-experimental | | Sample Size: *n=*38  *n*(Intervention) = 22  *n*(Comparison) = 16  Age Range: 12-18 years  Mean Age (SD): 14.9 (2.55)  Intervention = 14.61 (3.1)  Comparison = 15.49 (1.35)  Gender (Female): 71%  Mental health concerns: Moderate to severe depressive symptoms  Ethnicity: 2% Indigenous  CALD: 100% born in Australia  LGBTQIA+ identity: NR  Family socioeconomic status: NR | Acceptance and Commitment Therapy (ACT):  Treatment comprised of individual sessions following the ACT treatment manual. ACT is a form a psychotherapy that uses acceptance and mindfulness-based strategies as well as commitment and behaviour change strategies. Clinicians were trained in ACT and supervised by a clinical psychologist experienced in ACT.  Community setting: community mental health clinic | | Treatment as usual: Psychotherapy comprising of manualised cognitive behavioural therapy, which included psychoeducation, early warning signs planning, coping with unpleasant thoughts, increasing pleasurable activities, problem solving, goals setting, and crisis management. | NR |
| Hickey (2020), Victoria | Pre-post | | Sample Size: *n* = 18  Age Range: 15-25 years  Mean Age (SD): NR, 47% aged 15-17 years, 53% aged 18-25 years  Gender (Female): 59%  Mental health concerns: Young people who had a psychotic episode (71%) or ‘at risk mental state’ (29%). A large majority had comorbid major depression (59%), panic disorder (47%), or social anxiety (30%).  Ethnicity: NR  CALD: NR  LGBTQIA+ identity: n=2 (12%) non-binary/gender queer  Family socioeconomic status: NR | Mindfulness and Compassion Program for Psychotic Experiences:  The manualised mindfulness and compassion group intervention was facilitated by two experienced clinicians with mindfulness or meditation training. The program focused on teaching mindfulness skills to help participants to pay attention and recognize the transient nature of their experiences. Next, an emotion regulation model was introduced that focused on the interplay between a threat, motivational, and safety systems. The model helps participants understand the nature of their psychosis, anxiety and/or depression and how they are currently coping. Finally, compassion was introduced to help participants engage more skilfully with difficulties as they arise. Participants were encouraged to establish a regular mindfulness and compassion practice.  Community setting: Orygen centres - community mental health clinic | | Not applicable. | 8 weeks duration, one 90-minute session per week. |
| Hides (2011), Victoria | Quasi-experimental | | Sample Size: *n*=88  *n*(Intervention): 60  *n*(Comparison): 28  Age Range: 16-25 years  Mean Age (SD): 19.2 (1.6)  Intervention = 19.1 (1.4)  Comparison = 19.5 (2.0)  Gender (Female): 37%  Mental health concerns: Comorbid depression and substance use  Ethnicity: NR  CALD: NR  LGBTQIA+ identity: NR  Family socioeconomic status: two thirds of participants were receiving government benefits; 64% were unemployed | Cognitive Behavioural Therapy (CBT) and Motivational Interviewing (MI) Combined:  In addition to standard care, young people received sessions of CBT and MI delivered by a clinical psychologist. Support was driven by each client’s individual case formulation, incorporating CBT and MI strategies with psychoeducation.  Community setting: community mental health clinic, outreach | | Standard Care:  Case management plus brief motivational interviewing support for substance misuse delivered by an alcohol and other drug worker via outreach support. | Varied across participants, maximum of 12 x weekly sessions (average duration not recorded) |
| Hopkins (2017), Victoria | Pre-post | | Sample Size: *n*=43  Age Range: 15-25 years  Mean Age (SD): NR  Gender (Female): NR  Mental health concerns: High prevalent mental health concerns e.g., anxiety, depression, self-harm/suicidality.  Ethnicity: NR  CALD: NR  LGBTQIA+ identity: NR  Family socioeconomic status: NR | Single Session Family Therapy (SST):  SST aimed to provide clinical care to the young person and support families and carers to understand their young person’s experience and enable them to provide ongoing support at home. SST was delivered at Headspace; clinicians engaged the young people, parents and families either at their first point of contact with Headspace or via referral from their Headspace clinician. Sometimes, additional clinicians participated in the session from behind a screen, utilising a reflective team approach to provide additional insights on how the family was communicating. A session summary detailing issues and solutions was given to families and a follow-up phone call 4–5 weeks later to assess need for further support.  Community setting: Headspace centres - community mental health clinic | | Not applicable | Single session, duration not specified |
| Hudson (2015), New South Wales | Pre-post | | Sample Size: *n*=842  Age Range: 6-18 years  Mean Age (SD): 10.21 (2.57)  Gender (Female): 48%  Mental health concerns: Any anxiety disorder (51% generalised anxiety disorder, 21% social anxiety, 14% separation anxiety, 6% obsessive compulsive disorder, 7% specific phobia, 1% post-traumatic stress disorder).  Ethnicity: 63% Australian/Oceanic; 12% European; 5% Asian; 5% other not specified  CALD: NR  LGBTQIA+ identity: NR  Family socioeconomic status: NR | The Cool Kids program:  A manualised Group Cognitive Behavioural Therapy program designed for the management of broad-based childhood anxiety disorders. It included affect recognition, cognitive restructuring, child management, social skills training, assertiveness, and gradual exposure. Exposure tasks were therapist-designed in collaboration with the family to be specific to the child's needs. With the exception of one therapist assisted group session of in-vivo exposure, children completed gradual exposure under the direction of their parents for homework. The therapist monitored between session practice at the beginning of each session. Each group contained between 4 and 8 children with a range of different anxiety disorders. Both parents were encouraged to attend each session as well.  Community setting: University clinic | | Not applicable | 9-12 weekly sessions |
| Kehoe (2014), Victoria | Randomised controlled trial | | Sample Size: n=224  Age Range: 10-13 years  Mean Age (SD): 12.01 (0.42)  Gender (Female): 51%  Mental health concerns: Youth experiencing externalising mental health concerns  Ethnicity: 90% White  CALD: 18% did not speak English as first language – 8% European, 8% Asian, and 2% African languages  LGBTQIA+ identity: NR  Family socioeconomic status: 80% of parents in paid employment; 76% completed high school, 44% completed bachelor’s degree or higher; 39% had gross annual combined family income of $100,000 or more, 32% between $60,000-$99,000 | Tuning into Teens:  Tuning into Teens is a manualised 6-session group parenting program that teaches parents how to respond to their teen's emotions in ways that aim to increase communication and connection while helping the adolescent to understand their emotions. Parents were taught strategies for understanding and managing their own emotions and were educated about adolescent emotional development. The intervention was delivered through didactic teaching, role plays, exercises, home activities, and a DVD. Group sessions were conducted at a local community centre, with 6-13 participants per group. Primary facilitators were psychologists.  Community setting: community centre | | Control:  Parents did not receive Tuning into Teens. No intervention provided to control parents. | 6 weeks duration, one 2-hour session per week. |
| Poole (2018), Victoria | Randomised controlled trial | | Sample Size: *n*=64  *n*(intervention) = 31  *n*(control) = 33  Age Range: 12-18 years  Mean Age (SD): 15.2 (1.4)  Gender (Female): 73%  Mental health concerns: Depressive disorder (major, minor or dysthymic)  Ethnicity: NR  CALD: NR  LGBTQIA+ identity: NR  Family socioeconomic status: 12% of parents completed high school, 63% had tertiary education; parental income = 19% less than $20,000, 36% between $20,000-50,000, 21% between $50,000-80,000, 24% more than $80,000 | BEST MOOD program:  The BEST MOOD program is a manualised multi-family systems group therapy intervention focused on parent-child communication, stress reduction, psychoeducation and elements of attachment theory. BEST MOOD was delivered in community mental health settings by psychologists, or post-graduate clinical psychology trainees. The first four sessions were exclusively for parents (i.e., strategies for parents to engage their adolescent in the program, stress reduction techniques, child and family development and family unity, parent-child communication and parental self-care), with young people and their siblings invited to attend from week five through to eight (i.e., clarifying family roles, addressing major losses and trauma, enhancing patterns of communication, behavioural activation techniques, and promoting positive family rituals).  Community setting: across several community settings, not specified | | Parenting Adolescents Support Training (PAST):  A treatment-as-usual group program, with its content designed to reflect standard practice in currently available child and family services in Australia. It used supportive counselling techniques to facilitate therapeutic discussion between parents. All sessions were for parents, with young people invited to attend the fifth session only. | Eight weeks duration, weekly 2-hour sessions |
| Porter (2016), Western Australia | Pre-post | | Sample Size: *n*=153  Age Range: 12-16 years  Mean Age (SD): 13.6  Gender (Female): 29%  Mental health concerns: Severe and complex mental health concerns, at risk of out-of-home care placement or involvement in juvenile justice system  Ethnicity: 11% Aboriginal  CALD: NR  LGBTQIA+ identity: NR  Family socioeconomic status: NR | Multi-Systemic Family Therapy (MST):  MST provides intensive family and community-based treatment, focused on identifying aspects of the system in which a young person is embedded, and that when altered, increase the probability of reducing antisocial behaviour. MST is highly intensive, with a therapist having a concurrent caseload of only four to six families whom they typically visit three times each week in the family home. The assessment and intensive treatment were mostly home-based. After-hours telephone support was also available. MST interventions are evidence-based, utilising a range of empirically validated treatments that include cognitive behaviour therapy, parental skills training, and structural family therapy, whilst drawing on family systems theory and social ecological theories of behaviour.  Community setting: homes | | Not applicable | 4-6 months duration, approximately three contacts per week, clinicians are available 24/7 |
| Schley (2018), Victoria | Pre-post | | Sample Size: *n*=122  Age Range: 12-25 years  Mean Age (SD): 16.9 (3.35)  Gender (Female): 38%  Mental health concerns: Young people attending Headspace centres with general mental health concerns or mild to moderate levels of mental disorder, low-risk and limited complexity  Ethnicity: NR  CALD: NR  LGBTQIA+ identity: NR  Family socioeconomic status: NR | headspace Brief Intervention Clinic:  Young people received treatment delivered by a post-graduate trained allied health clinician. Treatment was based upon eight core modules, including communicating with confidence (supporting the development of assertive communication), exercise for wellbeing (psychoeducation and behavioural activation), problem solving skills, understanding and accepting self (acceptance and commitment framework for improving body image), understanding anger (anger management), understand my moods and anxiety (for anxiety and depression), mindfulness sand relaxation, and sleep.  Community setting: Headspace centres - community mental health clinic | | Not applicable | Approximately six weeks duration, weekly one-hour sessions |
| Tan (2015), Queensland | Randomised controlled trial | | Sample Size: *n*=91  *n*(Intervention) = 46  *n*(Comparison) = 45  Age Range: 13-18 years  Mean Age (SD): 15.4 (1.55)  Gender (Female): 75%  Mental health concerns: Primary psychiatric diagnosis (specific diagnoses not reported) and no prior mindfulness training, recruited from community child and adolescent mental health clinics  Ethnicity: NR  CALD: NR  LGBTQIA+ identity: NR  Family socioeconomic status: NR | Taming the Adolescent Mind:  Young people received treatment as usual plus five weekly group sessions (4-12 in each group). It aimed to enhance self-management of regulating attention to improve emotion regulation by promoting mindfulness, decentring from problematic thoughts and emotions, and increasing socioemotional awareness or resiliency through the use of simple sensory exercises e.g., use of visual and tactile exercises of ‘describing’, ‘judging’ and directing attention. Sessions were focused on: learning to regulate attention (mindful eating, psychoeducation, deep breathing), observer of present moment (skills of observing, mindful listening and visualisations) acceptance (body scan, mindful drawing activities, noticing without judgement), being mindful (consolidating mindful exercises and practice, accepting both positive and negative experiences, and mindfulness and beyond (implementing mindfulness in everyday life).  Community setting: community centre | | Treatment as usual: Standard care according to Axis 1 diagnoses in the International Classification of Diseases, comprising medication, family therapy, play therapy, and manualised cognitive behavioural therapy, which included psychoeducation, relapse prevention, coping with unpleasant thoughts, increasing pleasant activities, problem solving skills, crisis management, and goal setting. | 5 weeks duration, 5 weekly group sessions |
| Wagner (2017), Victoria | Quasi-experimental | | Sample Size: *n*=158  *n*(intervention) = 79  *n*(control) = 79  Age Range: 6-18 years  Mean Age (SD): 12.67  Gender (Female): 57%  Mental health concerns: Various mental health concerns (32% depression, 15% anxiety, 15% conduct or oppositional defiant disorder, 13% adjustment disorder, 7% acute stress disorder, 7% borderline personality disorder, 7% anorexia nervosa)  Ethnicity: NR  CALD: NR  LGBTQIA+ identity: NR  Family socioeconomic status: NR | Brief Intervention Therapy:  Brief intervention was offered within the local child and youth mental health services. Young people were provided with up to six sessions of psychological therapy from trainee psychologists. Each session was approached as though it may be the only one, and structured as a single session with a stated goal. Intervention incorporated elements of cognitive behavioural therapy and typically in- volves a solution-focused approach, including identifying both past family successes, and noting exceptions when the presenting problem did not occur. Client and family strengths were also elicited, with the focus being on tasks and strategies aimed to increase client and family efficacy. The Brief Intervention ‘dose’ is generally less than that of treatment as usual.  Community setting: combination of outreach (not specified) and centre-based | | Treatment as Usual:  Treatment as usual included case management incorporating assessment and psychological treatment (e.g., individual, parent, family therapy), plus linkage to other services. This generally occurs over a longer period of time than brief intervention. | Up to six 60-90-minute sessions over a three-month duration |
| Westwater (2020), Tasmania | Pre-post | | Sample Size: *n*=52  Age Range: 6-17 years  Mean Age (SD): 13.14 (2.96)  Gender (Female): 67%  Mental health concerns: High prevalent mental health concerns e.g., anxiety, depression, self-harm/suicidality  Ethnicity: NR  CALD: NR  LGBTQIA+ identity: NR  Family socioeconomic status: NR | Single Session Family Therapy (SSFT):  SSFT was delivered to youth engaged in a regional child and adolescent mental health service. SSFT is grounded in the belief that clients and families can effect change after one therapeutic encounter, using their own resources, with brief support and assistance from therapists. Two therapists were in the room with the family, while a one-way mirror with a multidisciplinary team of two to three clinicians reflecting behind was used. SSFT adopted a systemic approach by including the family members.  Community setting: community mental health clinic | | Not applicable | One session, duration not specified |
| **Case Management Programs** | | | | | | | |
| **First Author (Year), State** | **Study Design** | | **Participant**  **Characteristics** | **Intervention**  **Description** | | **Comparison Description** | **Duration and Frequency** |
| Brewer (2015), Victoria | Pre-post | | Sample Size: *n* = 170  *n*(Intervention) = 120  *n*(Comparison) = 50  Age Range: 15-24 years  Mean Age (SD):  Intervention = 20.9 (2.8)  Comparison = 20.1 (3.2)  Gender (Female): 25%  Mental health concerns: Young people with first-episode psychosis at-risk of harming themselves or others, 41% with comorbidity (e.g., personality disorder, polysubstance dependence).  Ethnicity: 72% white; 11% Asian; 9% African; 4% Middle Eastern; 3% Latino; 1% Maori; 1% Polynesian; 0% Aboriginal  CALD: NR  LGBTQIA+ identity: NR  Family socioeconomic status: NR | The Early Psychosis Prevention and Intervention Centre (EPPIC) Program – Intensive Case Management  EPPIC provided comprehensive treatment that is focused on the early detection, prevention, and intervention of psychosis. The pilot intensive case management subprogramme provided assertive outreach to young people experiencing first-episode psychosis. Intensive case management was distinguished from generic case management models by smaller caseloads, more client contact, greater coordination of services, multidisciplinary intervention, and greater emphasis on outreach, including provision of services in-house.  Community setting: assertive outreach - locations not specified | | EPPIC Treatment as Usual:  Matched controls received standard case management services from EPPIC.  Control group only used to compare hospital admission rates – all other outcomes assessed via pre-post. | Average duration of 10.7 (4.9) months, with an additional 13 months of treatment as usual |
| Chia (2013), Victoria | Pre-post | | Sample Size: *n*=59  Age Range: 11-17 years  Mean Age (SD): NR, mode = 15  Gender (Female): 68%  Mental health concerns: Young people with severe mental health concerns who are difficult to engage or at severe risk of harm (35% mood disorders, 22% anxiety disorders, 14% psychotic disorders, 47% previous suicide attempt, 59% rated as danger to self at time of referral, 66% comorbid diagnoses).  Ethnicity: NR  CALD: NR  LGBTQIA+ identity: NR  Family socioeconomic status: NR | The Adolescent Intensive Management (AIM) Team:  A multidisciplinary assertive outreach team embedded within the Austin Child and Adolescent Mental Health Service. The team combined intensive and flexible service delivery (i.e., outreach) with a broad range of intervention depending on each adolescent’s complex needs. Broad aims include engagement, improving young people’s developmental trajectory, overall functioning, and optimising family functioning. It aimed to address the developmental, psychodynamic, systemic, and neurobiological aspects of mental illness. Key interventions included individual therapy, crisis management, systemic therapy, and secondary consultations. Contact was as regular as several times a week, with after-hours telephone support provided if needed.  Community setting: assertive outreach - home, other locations not specified | | Not applicable | Average duration of 10.5 (6.8) months, varied frequency (up to several times a week if needed) |
| Conrad (2017), New South Wales | Pre-post | | Sample Size: *n*=1997  Age Range: 12-25 years  Mean Age (SD): 19.2 (4.5)  Gender (Female): 41%  Mental health concerns: High-risk mental health concerns, including a recent psychotic episode or those at risk of experiencing a psychotic episode based on current symptoms and impaired functioning.  Six baseline diagnostic groups were identified:  A: Existing psychosis  B: Recent psychosis  C: Ultra-high risk  D1 (lowest-risk group): Non-psychosis mental health disorders (no mental health admissions)  D2: Non-psychosis mental health disorders (prior mental health admissions)  E: Undetermined  Ethnicity: NR  CALD: NR  LGBTQIA+ identity: NR  Family socioeconomic status: NR | Psychological Assistance Service (PAS):  A specialised community-based mental health service designed for young people who have experienced psychosis. Staff conducted a preliminary risk assessment during an initial session with the young person, followed by more intensive assessments across subsequent sessions to determine the young person’s ongoing level of risk. A multidisciplinary team of clinicians provided ongoing mental health treatment, which includes psychiatric assessments, medication (if necessary), case management, cognitive behavioural therapy, family interviews, and other therapeutic treatment if appropriate.  Community setting: combination of assertive outreach and centre-based support at community mental health clinic | | Not applicable.  Outcomes were compared between the six diagnostic groups. | Duration not reported, frequency as needed based on the young person’s needs. |
| Daubney (2021), Queensland | Pre-post | | Sample Size: *n*=243  Age Range: 13-19 years  Mean Age (SD): 15.71 (1.45)  Gender (Female): 65%  Mental health concerns: Adolescents with severe and persistent mental health problems, who exhibited high risk behaviour, and were difficulty to engage in mainstream community services (47% neurotic, stress-related and somatoform disorders; 14% behavioral and emotional disorders; 10% mood disorders; 10% personality disorders; 5% substance use disorder; 4% developmental; 3% schizophrenia).  Ethnicity: 12% Aboriginal and/or Torres Strait Islander; 7% other (Maori, South African, or Papua New Guinea)  CALD: NR  LGBTQIA+ identity: 1% intersex, 7% reported gender incongruence, 15% same-sex attracted or bisexual  Family socioeconomic status: NR | Assertive Mobile Youth Outreach Service (AMYOS):  AMYOS provided assertive community treatment via mobile outreach (i.e., parks, home visits, cafés, schools, or ‘in-transit’) with flexible and frequent contact as required. Clinicians have a minimum four-year degree in psychology, social work, or mental health nursing, and at least five years of experience in community youth mental health. Clinicians conducted a six-week comprehensive assessment with the adolescent and family (where relevant) and implemented the developed treatment plan over a period of 12-18 months. Adolescents were provided with therapy (mentalisation-based integrated therapy) and practical support, including flexible practical assistance as required, such as attending Centrelink or medical appointments, re-engaging with school staff or managing problems with family and friends. Clinicians had a small caseload of up to 8 clients at a time to facilitate flexible, intensive, and tailored support, with out of hours care available.  Community setting: assertive outreach - homes, local community meeting points etc | | Not applicable | Average duration of 14 months, varied frequency of contact, as needed. |
| Howe (2017), New South Wales | Pre-post | | Sample Size: *n*=122  Age Range: 12-17 years  Mean Age (SD): NR  Gender (Female): 58%  Mental health concerns: Young people with moderate to severe mental illness  Ethnicity: NR  CALD: NR  LGBTQIA+ identity: NR  Family socioeconomic status: NR | Children and Young People’s Mental Health (CYPMH) Services:  A community-based specialist tertiary service for children and young people, embedded in the principles of early intervention, improving early access, integrated and collaborative support, and is youth friendly. It consisted of three components: Consultation and Assessment Team (point of access into service and assessment), Brief Intervention Team (short term intervention through care packages for up to 8 weeks), and the Youth Mental Health Team (clinicians have caseloads of 15-20 and use an assertive intensive case management approach). There is a focus on outreach to engage and holistic assessment, treatment and recovery.  Community setting: assertive outreach – predominantly homes | | Not applicable | Varied across participants |
| Killackey (2017), Victoria | Pre-post | | Sample Size: *n*=19  Age Range: 15-20 years  Mean Age (SD): 17.5 (1.0)  Gender (Female): 63%  Mental health concerns: Young people with severe mental illness, including first-episode psychosis, or mood disorder  Ethnicity: NR  CALD: 16% not born in Australia, not specified  LGBTQIA+ identity: NR  Family socioeconomic status: NR | Individual Placement and Support for Education (IPSed):  IPSed was delivered as part of Orygen’s Youth Health Clinical Program, at a community mental health clinic. An education specialist (trained teacher) aimed to link young people engaged in the service back to mainstream and alternative education settings. The specialist collaborated with the young person to help them re-engage in educational enrolment or identify educational courses that they felt matched their educational goals, prepared for enrolment and supported them while in the course, (i.e., transportation to the course, support in the classroom, assistance with homework etc.). The educational specialist liaised with school staff and the client’s case manager, and clinical care continued throughout.  Community setting: assertive outreach - homes, during transportation, parks | | Not applicable | Six months duration, frequency as needed |
| Killackey (2019), Victoria | Randomised controlled trial | | Sample Size: *n*=146  *n*(intervention) = 73  *n*(comparison) = 73  Age Range: 15-25 years  Mean Age (SD): 20.4 (2.4)  Gender (Female): 31%  Mental health concerns: Young people with first-episode psychosis (64% schizophrenia, 19% schizoaffective disorder; 17% major depressive disorder with psychotic features). High levels of comorbidity, including: 47% anxiety disorder, 43% substance use disorder, 20% bipolar disorder, 19% post-traumatic stress disorder.  Ethnicity: NR  CALD: 14% not born in Australia, not specified  LGBTQIA+ identity: NR  Family socioeconomic status: 90% of participants receiving government payments | Individual Placement and Support (IPS):  IPS was delivered as an addition to Orygen’s Early Psychosis Prevention and Intervention Centre (EPPIC), at a community mental health clinic. A vocational specialist was embedded as a member of the clinical team. Vocational specialists assist and support young people to gain employment or education. Intensity, frequency, and type of support was dependent upon each young person’s individual psychosocial needs and goals, such as support to remain engaged in school, study, training, find casual employment, improve job searching or application skills etc.  Community setting: combination of assertive outreach and centre-based support | | Treatment as usual:  Referral to external government-contracted employment agencies. All participants continued to receive standard EPPIC treatment, including medical management and review, outpatient case management, and peer support. | Six months duration, frequency as needed |
| Sabbioni (2018), Western Australia | Pre-post | | Sample Size: *n*=40  Age Range: 13-24 years  Mean Age (SD): 17 (2.8)  Gender (Female): 45%  Mental health concerns: Aboriginal youth with a serious mental health disorder who experience significant barriers to accessing mainstream mental health services (45% anxiety disorder, 30% mood disorder, 10% mixed conduct and emotional disorder, 2.5% schizophrenia, 60% co-occurring psychological disorders)  Ethnicity: 100% Aboriginal  CALD: 100% born in Australia  LGBTQIA+ identity: NR  Family socioeconomic status: NR | The YouthLink Model:  Provides recovery-oriented and evidence-based treatment for young people with complex mental health and psycho-social-cultural needs. It provides psychosocial and psychotherapeutic interventions, which are responsive to both the individual young person and to the systems within which they live. YouthLink provides a flexible approach to engagement with young people, with outreach capacity and assertive follow-up. Multidisciplinary team (including clinical psychology, social work, psychiatry and clinical nursing) and collaborative partnerships are developed between case managers, teams and health and community services, to provide person-centred care and a range of interventions including psychoeducation, supportive counselling and evidence-based psychological therapies. The team also includes two Aboriginal mental health practitioners who provide direct clinical services to, cultural consultation to non-Indigenous clinicians and community triage to support referrals.  Community setting: assertive outreach – homes, other locations not specified | | Not applicable | Varied across participants (dependent on need) – Average duration was 10.4 months and 11.4 sessions |
| Schley (2012)  Victoria | Pre-post | | Sample Size: *n*=44  Age Range: 15-25 years  Mean Age (SD): NR, median = 16.5  Gender (Female): 80%  Mental health concerns: Young people presenting with both psychotic and non-psychotic disorders who are considered ‘high-risk’  Ethnicity: NR  CALD: NR  LGBTQIA+ identity: NR  Family socioeconomic status: NR | Orygen’s Intensive Mobile Youth Outreach Services:  Young people received assertive outreach mental health care by a multidisciplinary team of clinicians and mental health professionals. Intervention components included comprehensive biopsychosocial assessment, practical support (e.g., finding suitable accommodation, securing employment, financial planning, etc.), psychotherapy, pharmacotherapy, risk management, family interventions, and system interventions (i.e., collaboration with other services that are involved). Intervention approaches were flexible and built around the client’s changing needs. Young people also had access to a Youth Access Team for support and emergency response outside of business hours.  Community setting: assertive outreach - homes, parks, or cafes | | Not applicable | Varied across participants (dependent on need) |
| **Integrated ‘one-stop-shop’ Programs** | | | | | | | |
| **First Author (Year), State** | **Study Design** | | **Participant**  **Characteristics** | **Intervention**  **Description** | | **Comparison Description** | **Duration and Frequency** |
| Bassilios (2017), National | Quasi experimental | | Sample Size: *n*=37,493  *n*(Intervention): 17,337  *n*(Comparison): 20,156  Age Range: 12-25 years  Mean Age: NR  Gender (Female): 65%  Mental health concerns: Mild to moderate mental health concerns  Ethnicity: 5% Indigenous; 79% not Indigenous; 16% unknown  CALD: NR  LGBTQIA+ identity: NR  Family socioeconomic status: 23% considered ‘low-income’ | Headspace:  Headspace provided highly accessible, youth-friendly, integrated service hubs and networks that provide free or low-cost evidence-based intervention and support to young people. Headspace provided young people with access to several in-house services, in addition to referrals for complementary community-based services, including predominantly psychological services (individual and group therapy), as well as additional health and biopsychosocial services, such as physical and sexual health, alcohol or other drug support, and vocational services.  Community setting: Headspace centres – community mental health clinic | | Allied Psychological Services:  Provision of primary care for high prevalent disorders (e.g., depression and anxiety). Psychologists provide free or low-cost evidence-based mental health care for up to 12 individual sessions and 12 group sessions per calendar year. | Varied across participants, not specified |
| Goel (2021), Western Australia | Pre-post | | Sample Size: *n =* 308  Age Range: 16-24 years  Mean Age (SD): 18.2 (2.1)  Gender (Female): 62%  Mental health concerns: Acute or complex mental health conditions, with a trauma history (25% mood disorder; 17% neurotic, stress-related, and somatoform disorders; 34% other or unspecified; 8% substance use disorder; 8% schizophrenia; 8% personality disorder  Ethnicity: 96% White; 2% Aboriginal and/or Torres Strait Islander; 2% - Somali, Asian, or Maori  CALD: NR  LGBTQIA+ identity: 3% transgender or gender diverse  Family socioeconomic status: NR | Youth Community Assessment and Treatment Team (YCATT):  YCATT consisted of a multidisciplinary team of psychiatrists, nurses, social workers, pharmacists, teachers, aboriginal liaison officers, and clinical psychologists. Young people received a comprehensive assessment and management plan. The psychiatrist provided regular medical reviews. Young people received 4–6 sessions of cognitive-behavioural therapy from the psychologist, and psychoeducation, problem solving, motivational interviewing, supportive psychotherapy, or crisis support from the case manager. The social worker addressed any challenges with housing, referrals to disability services, obtaining financial support etc. Teachers liaised with the school/college to address concerns regarding attendance or performance and prepared behaviour management plans. Sessions were primarily conducted at home, outpatient clinic, or other community settings. Family members were allowed to attend any appointments.  Community setting: community mental health clinic with opportunity for outreach | | Not applicable | Mean duration of 61 days (SD=44 days), intended duration was 6-8 weeks.  Frequency ranged from 1-2 times per week to daily contact. |
| Green (2015), Victoria | Pre-post | | Sample Size: *n*=42  Age Range: 12-24 years  Mean Age (SD): 16.66 (2.86)  Gender (Female): 100%  Mental health concerns: Anorexia nervosa (83% restricting type, 17% binge eating/purging type). High rate of comorbidity, including depressive disorder (69%), anxiety disorder (53%) and borderline personality disorder (6%)  Ethnicity: NR  CALD: NR  LGBTQIA+ identity: NR  Family socioeconomic status: NR | Butterfly Eating Disorder Day Program:  The program was staffed by a multi-disciplinary team, including a consultant psychiatrist, paediatrician, teacher, five mental health clinicians, an art therapist, and an administrative assistant. Each participant was assigned a key support worker, who coordinated their program care. The cognitive behavioural group therapy component ran five days per week, between 9:30am and 3:15pm. Clinicians were also available for individual, dietetic and family sessions outside of group hours. Participants ate morning tea, lunch and afternoon tea as a group facilitated by two staff members, and were weighed weekly, with their weight determining their individual meal plan. To assist with relapse prevention and re-integration into the community, participants brought their own food each day from home, reduced to part-time program participation prior to discharge, and developed a relapse prevention plan with their key support worker.  Community setting: community mental health clinic | | Not applicable | Five days per week, for two to six months duration, dependent on participant need. |
| Kennair (2011), Victoria | Quasi-experimental | | Sample Size: *n*=84  Age Range: 12-18 years  Mean Age (SD): NR  Gender (Female): NR  Mental health concerns: Young people who are experiencing moderate to severe mental health concerns  Ethnicity: NR  CALD: NR  LGBTQIA+ identity: NR  Family socioeconomic status: NR | Adolescent Day Program:  An integrated therapeutic and vocational non-residential outpatient group program for a group of 6-8 adolescents at a time. Adolescents were referred by their outpatient Child and Adolescent Mental Health Services (CAMHS) Case Manager, who continued to provide case management (see comparison group). The program included a structured timetable of five sessions, such as skills-based work (social skills training, anger management), flexible psychotherapeutic group work, and outings. Each young person was allocated a key worker who acts as their contact person and spends 45 minutes per week with them to help to achieve their psychosocial goals, and liaises with other services involved in the young person’s care.  Community setting: community mental health clinic | | Outpatient treatment with CAMHS:  Young people received standard case management from clinicians within a multidisciplinary team (i.e., psychologist, psychiatrist, social worker, occupational therapist, and psychiatric nurse). Treatment may include assessment, psychoeducation, medication, and a range of therapies. | Intervention:  9 weeks duration – 3x5-hour sessions per week  Comparison:  6 months – 1 session per week (session duration N/R) |
| Klag (2021), Queensland | Pre-post | | Sample Size: *n* = 664  Age Range: 7-14 years  Mean Age (SD): 10.6  Gender (Female): 38%  Mental health concerns: Children in out-of-home care with complex and severe mental health concerns, high rate of comorbidity (49% attachment disorders, 21% post-traumatic stress disorder, 18% mood disorders, 17% conduct disorders, 17% ADHD, 15% emotional and behavioural disorders, 8% anxiety).  Ethnicity: 27% Aboriginal and/or Torres Strait Islander  CALD: NR  LGBTQIA+ identity: NR  Family socioeconomic status: NR | Evolve Therapeutic Service (ETS):  ETS is a trauma-informed wrap-around model of care that aims to provide coordinated therapeutic and behaviour supports to young people in out-of-home care. ETS teams are situated within Child and Youth Mental Health Services, and comprise of a multidisciplinary team of psychologists, social workers, nurses, and occupational therapists. ETS uses evidence-informed individual and systemic therapeutic interventions. Clinical interventions include a comprehensive assessment, and attachment and/or trauma focused therapies (i.e., dyadic work, individual therapy, family-based intervention). Systemic interventions involve carers, biological parents, educational staff and other professionals involved. This includes assisting and facilitating the development of a regular cohesive stakeholder group to work collaboratively in the child's best interests, provision of carer support including foster carer training, specialist consultation-liaison services, and specialist professional development and training.  Community setting: community mental health clinic | | Not applicable | Average duration of 19.2 months, varied frequency based on need |
| Rickwood (2015), National | Pre-post | | Sample Size: *n*=20,034  Age Range: 12-25 years  Mean Age (SD): 17.8 (3.3)  Gender (Female): 63%  Mental health concerns: Mild to moderate mental health concerns (anxiety and depression)  Ethnicity: NR  CALD: NR  LGBTQIA+ identity: NR  Family socioeconomic status: NR | Headspace:  This included 55 participating Headspace centres across Australia, funded by the National Youth Mental Health Foundation. Headspace is a one-stop shop for mental health support that provides young people with access to a range of in-house services, in addition to referrals for complementary community-based services. Headspace centres provide care across several key domains, including mental health, physical health, substance use, and social and vocational participation. Centres provide early mental health intervention through psychotherapy delivered by psychologists.  Community setting: Headspace centres - community mental health clinic | | Not applicable | Varied across participants |
| **Lifestyle Programs** | | | | | | | |
| **First Author (Year), State** | **Study Design** | | **Participant**  **Characteristics** | **Intervention**  **Description** | | **Comparison Description** | **Duration and Frequency** |
| Curtis (2016), New South Wales | Quasi-experimental | | Sample Size: *n*=28  Age Range: 17-25 years  Mean Age (SD): 20.7 (2.2)  Gender (Female): 39%  Mental health concerns: Young people with first-episode psychosis (e.g., psychosis, schizophrenia, schizoaffective disorder, bipolar affective disorder, or depression with psychotic features) who were being treated with antipsychotic medication.  Ethnicity: 62% Caucasian; 25% Asian; 13% Indigenous  CALD: NR  LGBTQIA+ identity: NR  Family socioeconomic status: NR | The Keeping the Body in Mind (KBIM) Programme:  KBIM was delivered by a multidisciplinary team that consists of a clinical nurse consultant, a dietician, an exercise physiologist, a youth peer wellness coach, and an endocrinologist. Intervention components included health coaching delivered through motivational interviewing, weekly dietetic support (e.g., education surrounding weight management, nutrition, cooking skills, etc.), an individually tailored supervised exercise program, and peer wellness coaching (support from two young people with lived experience of psychosis).  Community setting: local community centres, Orygen community mental health clinic, | | Standard Care:  Participants received standard care which followed standard clinical guidelines, consisting of individual mental health case management alongside medical assessments and the prescription of antipsychotic medication. | 12 weeks (frequency not reported) |
| McGuire (2021), Victoria | | Pre-post | Sample Size: *n*=14  Age Range: 15-24 years  Mean Age (SD): 19.9 (3.11)  Gender (Female): 79%  Mental health concerns: Early psychosis with anxiety (28% schizophrenia, 24% psychotic disorder, 21% bipolar affective disorder with psychosis, 14% at ultrahigh risk for psychosis, 7% schizophreniform, 7% delusional disorder).  Ethnicity: NR  CALD: 7% born in India; 7% born in Sri Lanka  LGBTQIA+ identity: NR  Family socioeconomic status: NR | | Yoga intervention:  The yoga intervention was delivered within Orygen’s early intervention for psychosis service. Yoga classes were delivered by qualified yoga teachers. They delivered Slow Vinyasa yoga, which incorporated basic pranayama/breathing techniques, asana/postures and relaxation in each session. Sessions were all conducted in-person, on-site. Each session comprised of 30 minutes of low-moderate level physical activity and 20 min of pranayama and relaxation. Young people were taught to focus on their breath, to link breath with movement and were provided with instruction about the importance of breathing. Asana involved a combination of seated, standing, balancing and supportive or restorative postures.  Community setting: Orygen centres - community mental health clinic | Not applicable | Six weeks duration, weekly 50-minute sessions |
| Nasstasia (2017), New South Wales | Pre-post | | Sample Size: 12  Age Range: 15-25 years  Mean Age (SD): 20.83 (1.70)  Gender (Female): 75%  Mental health concerns: Major depressive disorder  Ethnicity: 92% Anglo Australian; 8% European  CALD: NR  LGBTQIA+ identity: NR  Family socioeconomic status: NR | Motivational Interviewing and Exercise Intervention:  An integrated multi-modal intervention that combined motivational interviewing and exercise. A single session (90-minute) of motivation interviewing intervention was provided to enhance participants’ motivation to engage in the exercise program. Personal trainers adopted motivational interviewing approaches and principles to build a working alliance, explore previous experiences with exercise, barriers to exercise and provide psychoeducation around the exercise-mood link. Then, participants engaged in a 12-week group multi-modal standardised exercise intervention at a gym at the university gym. The exercise program included resistance training, endurance, power and aerobic exercise. Participants exercised in small groups.  Community setting: University clinic and gym | | Not applicable | Three months duration, 3x1 hours sessions per week |
| Nasstasia (2019), New South Wales | Randomised controlled trial | | Sample Size: *n*=53  *n*(Intervention) = 23  *n*(Comparison) = 30  Age Range: 15-25 years  Mean Age (SD): 20.75 (2.59)  Gender (Female): 78%  Mental health concerns: Major depressive disorder  Ethnicity: NR  CALD: NR  LGBTQIA+ identity: NR  Family socioeconomic status: NR | Healthy Body Healthy Mind:  An integrated multi-modal intervention combining motivational interviewing and exercise. Firstly, a single session (90-minute) of motivation interviewing intervention was provided to enhance participants’ motivation to engage in the exercise program. Personal trainers adopted motivational interviewing approaches and principles to build a working alliance, explore previous experiences with exercise, barriers to exercise and provide psychoeducation around the exercise-mood link. Then, participants engaged in a 12-week group multi-modal standardised exercise intervention at a gym at the university gym. The exercise program included resistance training, endurance, power and aerobic exercise. Participants exercised in small groups.  Community setting: University clinic and gym | | Waitlist control: Participants were advised to not make any changes to their lifestyle until they commenced the intervention. | Three months duration, 3x1 hour sessions per week |
| Parker (2016), Victoria | Randomised controlled trial | | Sample Size: *n*=174  *n*(Intervention) = 88  *n*(Comparison) = 86  Age Range: 15-25 years  Mean Age (SD): 17.6 (2.4)  Intervention = 17.75 (2.58)  Comparison = 17.44 (2.29)  Gender (Female): 61%  Mental health concerns: Mood or anxiety disorders  Ethnicity: NR  CALD: NR  LGBTQIA+ identity: NR  Family socioeconomic status: NR | Physical activity intervention:  The intervention was based on behavioural activation principles, focusing on goal setting, creating opportunities to engage in physical activity, and monitoring the connection between mood, anxiety, and activity levels. Therapists provided psychoeducation on the relationship between exercise and mood/ anxiety symptoms, guidelines for physical activity, a costs and benefits worksheet about engaging in physical activity, physical activity diaries and pedometers for motivational purposes. The type of physical activity was not prescribed; physical activities were tailored and chosen based on the individual participant’s interests, prior activities that were enjoyable/offered a sense of achievement, current activity or perceived fitness levels, resources and social supports.  Community setting: Headspace centres – community mental health clinic | | Lifestyle psychoeducation intervention:  Participants received the same psychoeducation and resources as the treatment group as well as weekly resources focused on sleep, substance use and other lifestyle information. The resources were discussed in terms of the content, but therapists did not discuss how to act or apply the information. | 6 weeks duration, weekly one-hour sessions |
| Pearce (2020), Victoria | Pre-post | | Sample Size: *n*=312  Age Range: 15-25 years  Mean Age (SD): 19.8 (2.9)  Gender (Female): 51%  Mental health concerns: Any mental health disorder (59% first episode psychosis, 13% risk for psychosis, 12% mood disorder, 16% personality disorder)  Ethnicity: NR  CALD: 18% first generation migrants  LGBTQIA+ identity: NR  Family socioeconomic status: NR | Addition of an Exercise Physiology Service at Orygen:  The service involved a baseline assessment (including previous and current physical activity levels, and cardiorespiratory fitness) and the development of an individualised program. It was designed to balance the need to achieve sufficient activity intensity and volume that was challenging, with the need for the activity to be enjoyable. Some examples of individualised programmes included the exercise physiologist supervising weights training at a local gym or working with the young person on a body weight routine. Group programs were run at Orygen sites and involved gym, yoga or boxing group.  Community setting: Orygen centres - community mental health clinic, and local gyms or community centres for exercise, yoga, and boxing groups | | Not applicable | Not recorded |

*Note.* NR = not reported; CALD: culturally and linguistically diverse; LGBTQIA+: lesbian, gay, bisexual, transgender, queer, intersex, asexual or other

Supplementary Table 3*. Summary of Results*

| **Therapy Programs** | | | | | |
| --- | --- | --- | --- | --- | --- |
| **First Author (Year)** | **Assessment Time Points** | **Outcomes and Measures** | **Descriptive Statistics**^1^  **(M and SD)** | **Significance of Difference and Effect Size** | **Summary of Findings** |
| Edwards (2018) | Time 1: before group therapy  Time 2: post group therapy | A Before and After Survey was provided to participants to measure six feelings:   1. Relaxed 2. Anxiety 3. Motivation 4. Tiredness 5. Stress 6. Comfort | M and SD not reported, only whether mean scores increased or decreased:   1. Increased 2. Decreased 3. Increased 4. Increased 5. Decreased 6. Increased | Significance and effect sizes not reported and cannot be calculated. | No statistically significant differences due to very small sample size. |
| Farrell (2012) | Time 1: pre-treatment  Time 2: post-treatment (13 weeks)  Time 3: six months follow-up | 1. Diagnosis of obsessive-compulsive disorder (OCD): Anxiety Disorders Interview Schedule for Children 2. OCD symptoms: Children’s Yale-Brown Obsessive-Compulsive Scale 3. Severity of OCD symptoms: National Institute of Mental Health Global Obsessive-Compulsive Scale 4. Impact of OCD symptoms on psychosocial functioning: Child OCD Impact Scale | 1. Pre: 5.89 (1.92)   Post: 2.08 (2.28)   1. Pre: 20.45 (6.82)   Post: 12.43 (8.18)   1. Pre: 8.89 (1.92)   Post: 4.83 (2.44)   1. Pre: 29.00 (17.07)   Post: 16.17 (19.41) | 1. *t*=10.937, *p*<.01, *d*=1.65 2. *t*=5.93, *p*<.01, *d*=0.92 3. *t*=9.843, *p*<.01, *d*=1.65 4. *t*=3.056, *p*<.005, *d*=0.69 | The OCD group therapy program yielded significant reductions in the presence, severity, and psychosocial impact associated with OCD symptoms. |
| Hall (2021) | Time 1: baseline  Time 2: six-weeks post-intervention | 1. Emotion regulation: Difficulties in Emotional Regulation Scale (DERS-16) 2. Mental health symptoms: The Depression, Anxiety and Stress Scales (DASS-21) 3. Mindfulness: The Cognitive and Affective Mindfulness Scale | Not reported for the overall sample. | 1. *p*<.05 2. *p*<.05 3. *p*>.05   Effect sizes not reported and cannot be calculated | There were significant reductions in symptoms of depression, anxiety, and stress, and improvements in emotion regulation. No significant change in mindfulness. |
| Havighurst (2015) | Time 1: baseline  Time 2: follow-up (10.5 months post baseline) | 1. Family conflict: Family Conflict Scale (Parent-report) 2. Family conflict: Family Conflict Scale (Young person-report) 3. Externalising mental health concerns: Strengths and Difficulties Questionnaire (SDQ, Parent-report) 4. Externalising mental health concerns: SDQ (Young person-report) | 1. Intervention: 2.85 (0.20)   Control: 2.73 (0.21)   1. Intervention: 2.22 (0.19)   Control: 2.66 (0.20)   1. Intervention: 3.49 (0.30)   Control: 4.02 (0.32)   1. Intervention: 4.18 (0.30)   Control: 4.95 (0.32) | 1. *t*=-2.88, *p*=.004, *d*=.32 2. *t*=-1.98, *p*=.049, *d*=.25 3. *t*=-3.04, *p*=.003, *d*=.31 4. *t*=-2.18, *p*=.030, *d*=.28 | There were significant reductions in family conflict and externalising behaviour problems among youth in the intervention group, compared to the control group. |
| Hayes (2011) | Time 1: Pre-treatment  Time 2: six weeks post-treatment  Time 3: three months follow-up | 1. Depressive symptoms: Reynolds Adolescent Depression Scale 2. Emotional and behavioural functioning: SDQ | 1. Intervention: 56.92 (4.61)   Control: 85.22 (6.42)   1. Intervention: 11.37 (1.64)   Control: 20.72 (2.31) | 1. *p*<.001, *d*=-1.45 2. *p*<.01, *d*=-2.14 | There were significant improvements in emotional and behavioural functioning, particularly depressive symptoms among intervention participants. |
| Hickey (2020) | Time 1: baseline  Time 2: post-treatment (8 weeks)  Time 3: 6-week follow-up | 1. Self-compassion: Self-Compassion Scale (SCS) 2. Mindfulness skills: Five Facet Mindfulness Questionnaire (FFMQ) 3. Mental-state: Comprehensive Assessment of the At Risk Mental States (CAARMS) 4. Psychiatric symptoms: Brief Psychiatric Rating Scale (BPRS) 5. Depression, anxiety, and stress symptoms: DASS-21 6. Role functioning: Global Functioning Role Scales 7. Social functioning: Global Functioning Social Scales | 1. Pre: 2.51 (0.75)   Post: 2.83 (0.78)   1. Pre: 109 (16.9)   Post: 110 (16.7)   1. Pre: 44.07 (26.24)   Post: 37.29 (22.39)   1. Pre: 63.17 (12.32)   Post: 43.67 (7.34)   1. Not reported. 2. Pre: 5.29 (1.82)   Post: 6.71 (2.02)   1. Pre: 6.21 (1.31)   Post: 7.64 (1.49) | 1. *p*=.047, *d*=.563 2. *p*=.765, *d*=.078 3. *p*=.178, *d*=-.380 4. *p*=.005, *d*=-2.073 5. *p*<.001, *d*=-1.405 6. *p*<.001, *d*=1.164 7. *p*<.001, *d*=1.319 | There were significant increases in self-compassion, psychosocial functioning, and reduction in mental health symptomology among participants following the mindfulness and compassion program. |
| Hides (2011) | Time 1: baseline  Time 2: post-intervention (three months)  Time 3: six months | 1. Psychological distress: Kessler Psychological Distress Scale 2. Depressive symptoms: Hamilton Depression Rating Scale 3. Depressive symptoms: Centre of Epidemiologic Studies Depression Scale 4. Psychosocial functioning: Social and Occupational Functioning Assessment Scale 5. Proportion of participants with Substance Use Disorder Diagnosis: DSM-IV 6. Frequency of alcohol use: days of use 7. Frequency of cannabis use: days of use | 1. Intervention: 23.7 (1.1)   Control: 22.2 (1.5)   1. Intervention: 11.3 (0.8)   Control: 9.6 (1.1)   1. Intervention: 22.3 (1.8)   Control: 18.7 (2.5)   1. Intervention: 69.0 (1.4)   Control: 70.9 (2.0)   1. Intervention: 54%   Control: 79%   1. Intervention: 5.0 (1.1)   Control: 8.8 (1.5)   1. Intervention: 10.2 (1.6)   Control: 10.5 (2.2) | 1. *p*=0.498, *d*=1.14 2. *p*=0.292, *d*=1.77 3. *p*=0.731, *d*=1.65 4. *p*=0.588, *d*=1.10 5. *p*<.05, *d*=-.250 6. *p*=0.492, *d*=2.89 7. *p*=0.677, *d*=.156 | There was a significant reduction in the proportion of participants with substance used disorder among intervention participants compared to controls. All other outcomes were not statistically significant. |
| Hopkins (2017) | Time 1: before session  Time 2: 4-5 weeks after the session | 1. Psychosocial functioning: Outcome Rating Scales (ORS) | Not reported for overall sample | 1. *F*(1, 40)=18.55, *p*<.001   Effect sizes not reported and cannot be calculated | Young people had significantly improved functioning (i.e., individual, interpersonal, social role and overall functioning) following the session. |
| Hudson (2015) | Time 1: pre-treatment  Time 2: post-treatment  Time 3: 3-12 months follow-up | 1. Anxiety disorder diagnostic severity: Clinician Severity Rating 2. Anxiety symptom severity: Spence Children’s Anxiety Scale (SCAS) | Not reported for overall sample | 1. *F*(2, 1642)=716.03, *p*<.01 2. *F*(2, 1586)=162.84, *p*<.01   Effect sizes not reported and cannot be calculated | There was a significant reduction in the presence and severity of anxiety symptoms following treatment. |
| Kehoe (2013) | Time 1: Baseline  Time 2: Follow-up (10.5 months post baseline) | 1. Anxiety symptoms: (SCAS) 2. Anxiety symptoms: Spence Child Anxiety Scale for Parents (SCAS-P) 3. Depressive symptoms: Child Depression Inventory (parent-report) 4. Depressive symptoms: Child Depression Inventory (child-report) | 1. Intervention: 10.94 (0.85)   Control: 14.45 (0.93)   1. Intervention: 17.05 (1.35)   Control: 21.79 (1.45)   1. Intervention: 9.23 (0.48)   Control: 8.91 (0.44)   1. Intervention: 1.45 (0.27)   Control: 1.86 (0.29) | 1. *t*=-4.92, *p*<.001, *d*=.46 2. *t*=-2,17, *p*=.031, *d*=.26 3. *t*=-4.06, *p*<.001, *d*=.46 4. *t*=-1.17, *p*=.244, *d*=.14 | There was a significant reduction in parent and youth-reported anxiety, and parent-reported depressive symptoms among intervention participants, in comparison to the control group. |
| Poole (2018) | Time 1: baseline  Time 2: post-treatment (8 weeks)  Time 3: three-month follow-up | 1. Mental health difficulties: SDQ 2. Depressive symptoms: The Short Moods and Feelings Questionnaire (SMFQ) 3. Alcohol consumption The Alcohol Use Disorders Identification Test (AUDIT) | 1. Intervention: 19.45 (1.11)   Control: 18.64 (1.11)   1. Intervention: 13.32 (1.47)   Control: 14.11 (1.40)   1. Intervention: 2.13 (0.56)   Control: 1.69 (0.55) | 1. *F*(1, 40)=0.455, *p*=.50, *d*=.26 2. *F*(1, 44)=0.364, *p*=.55, *d*=.83 3. *F*(1, 35)=0.633, *p*=.43, *d*=-.16 | There were improvements over time in mental health symptoms, but no significant differences between intervention and control groups. |
| Porter (2016) | Time 1: baseline  Time 2: post-treatment (4-6 months)  Time 3: six-month follow-up  Time 4: 12-month follow-up | 1. Internalising mental health problems 2. Externalising mental health problems 3. Total problems   All outcomes were measured using the Child Behaviour Checklist (CBCL) | 1. Pre: 21.48 (11.63)   Post: 14.35 (10.57)   1. Pre: 38.18 (10.13)   Post: 24.07 (12.30)   1. Pre: 92.54 (28.09)   Post: 61.02 (30.65) | 1. *F*=61.10, *p*<.001, *η^2^*=.182 2. *F*=98.13, *p*<.001, *η^2^*=.313 3. *F*=105.94, *p*<.001, *η^2^*=.311 | Young people experienced significant reductions in internalising and externalising mental health symptoms following the intervention. |
| Schley (2018) | Time 1: Pre-treatment  Time 2: Post-treatment (6 weeks) | 1. Psychological distress: Kessler Psychological Distress Scale-10 (K-10) 2. Depressive symptoms: The Quick Inventory of Depressive Symptoms 3. Anxiety symptoms: The Overall Anxiety Severity and Impairment Scale 4. Psychosocial functioning: The Social and Occupational Functioning Assessment Scale | 1. Pre: 23.56 (7.92)   Post: 17.88 (7.16)   1. Pre: 10.46 (5.57)   Post: 7.00 (4.84)   1. Pre: 6.20 (4.35)   Post: 3.63 (4.07)   1. Pre: 69.9 (9.67)   Post: 75.6 (9.63) | 1. *t*(76)=21.89, *p*<.001, *d*=0.75 2. *t*(70)=12.18, *p*<.001, *d*=0.66 3. *t*(70)=7.51, *p*<.001, *d*=0.61 4. *t*(89)=74.5, *p*<.001, *d*=0.59 | There was significant improvements across all measures of psychiatric symptoms and psychosocial functioning among participants. |
| Tan (2015) | Time 1: baseline  Time 2: post-intervention (5 weeks)  Time 3: three months follow-up | 1. Psychological distress: DASS-21 2. Self-esteem: Rosenberg Self-Esteem Scale 3. Resilience: Resiliency Scale for Children and Adolescents 4. Mindfulness: Children’s Acceptance and Mindfulness Measure 5. Psychological inflexibility: Avoidance and Fusion Questionnaire for Youth 6. General psychological symptoms: CBCL | 1. Intervention: 44.33 (27.91)   Control: 65.78 (26.75)   1. Intervention: 16.07 (8.35)   Control: 10.86 (4.50)   1. Intervention: 38.81 (10.79)   Control: 31.49 (9.86)   1. Intervention: 22.79 (7.35)   Control: 16.24 (7.80)   1. Intervention: 12.14 (7.53)   Control: 17.78 (6.75)   1. Intervention: 57.60 (9.04)   Control: 67.46 (7.94) | 1. *F*(1,78)=16.51, *p*<.001, *η^2^*=.18 2. *F*(1,78)=10.85, *p*<.001, *η^2^*=.12 3. *F*(2,156)=55.75, *p*=.137, *η^2^*=.03 4. *F*(1,78)=34.49, *p*<.001, *η^2^*=.31 5. *F*(1,78)=18.73, *p*<.001, *η^2^*=.19 6. *F*(1,78)=10.84, *p*<.001, *η^2^*=.12 | There was significantly decreased psychological distress and improved self-esteem, mindfulness, and psychological functioning among intervention participants in comparison to controls. |
| Wagner (2017) | Time 1: after the first session  Time 2: after the final session (3 months) | 1. Mental health functioning: Health of the Nations Outcome Scale for Children and Adolescents (HoNOSCA) | 1. Intervention: 11.28 (6.35)   Control: 11.01 (7.68) | 1. *p*>.05, *d*=.038 | Improvements in mental health functioning was not significant between the treatment and control groups. |
| Westwater (2020) | Time 1: before session  Time 2: two weeks after the session | 1. Worry 2. Confidence 3. Child and adolescent mental health service engagement post session   All outcomes were measured using the Single Session Family Therapy Pre-Post Questionnaire | 1. Pre: 7.49 (2.87)   Post: 5.05 (2.78)   1. Pre: 4.40 (2.61)   Post: 5.45 (2.65)   1. 63% required no further engagement with CAMHS following SSFT | 1. *z*=-2.63, *p*=.01, *d*=.864 2. *z*=-1.75, *p*=.08, *d*=.399 3. Not applicable | SSFT yielded significant reductions in worry among youth, and 63% did not require further contact with CAMHS. There was no significant change in confidence level. |
| **Case Management Programs** | | | | | |
| **First Author (Year)** | **Assessment Time Points** | **Outcomes and Measures** | **Descriptive Statistics**^1^  **(M and SD)** | **Significance of Difference** | **Summary of Findings** |
| Brewer (2015) | Time 1: referral  Time 2: discharge (average 10 months) | 1. Number of inpatient admissions 2. Number of inpatient ward days 3. Psychiatric symptoms: BPRS 4. Negative symptoms: Scale for the Assessment of Negative Symptoms 5. Social functioning: Health of the Nation Outcomes Scales (HoNOS) 6. General functioning: Life Skills Profile | 1. Intervention: 0.65 (0.83)   Control: 2.30 (2.49)   1. Intervention: 8.23 (23.82)   Control: 27.98 (30.35)   1. Pre: 64.29 (13.06)   Post: 49.84 (8.96)   1. Pre: 23.33 (14.57)   Post: 13.58 (9.41)   1. Pre: 16.12 (8.73)   Post: 12.88 (8.57)   1. Pre: 18.22 (4.11)   Post: 18.62 (2.61) | 1. *U* = 2345.50, *p*<.05, *d*=.889 2. *U* = 2241.00, *p*<.05, *d*=.724 3. *t*(119) = 10.04, *p*<.001, *d*=1.29 4. *t*(119) = 6.14, *p<*.001, *d*=.795 5. *t*(119) = 3.79, *p*<.001, *d*=.375 6. *t*(119) = -0.88, *p*=.38, *d*=.116 | There was a significantly lower number of admissions and inpatient days among the intervention group compared to controls.  Psychiatric symptoms, negative symptoms, and social functioning significantly improved from referral to discharge. |
| Chia (2013) | Time 1: referral  Time 2: discharge (average 10 months) | 1. Number of hospitalisations: Proportion of sample with 0 admissions 2. Engagement in education/vocational training: Proportion not attending school 3. Psychosocial functioning: Children’s Global Assessment Scale (CGAS) | 1. Pre: 53%   Post: 83%   1. Pre: 52%   Post: 19%   1. Mean change = 9.1 (7.7) | 1. *p*<.005 2. *p*<.005 3. *p<*.001   Effect sizes not reported and cannot be calculated | There was significantly reduced readmission rates, increased school attendance and improved psychosocial functioning from service referral to discharge. |
| Conrad (2017) | Time 1: pre-treatment  Time 2: post-treatment | 1. Depression diagnosis 2. Anxiety diagnosis 3. Number of hospital admissions | Not reported | 1. *p*<.001 2. *p*<.001 3. *p*<.001   Effect sizes not reported and cannot be calculated | There was a significant reduction in the number of diagnoses and hopsital admissions following intervention. |
| Daubney (2021) | Time 1: pre-treatment, during assessment  Time 2: post-treatment (average 14 months) | 1. Mental health functioning: HoNOSCA 2. Suicidality: BPRS 3. General functioning: CGAS 4. High-risk behaviours: Frequency of Risk Behaviours 5. Child behaviour: SDQ 6. Engagement: The Engagement Measure 7. Emergency department presentations      1. Mental health-related hospital admissions 2. Length of stay in inpatient psychiatric unit: average monthly number of nights | 1. Pre: 21.53 (7.03)   Post: 14.97 (8.73)     1. Pre: 3.68 (1.53)   Post: 2.50 (1.41)   1. Pre: 43.37 (10.50)   Post: 59.66 (12.40)   1. Pre: 1.16 (0.99)   Post: 0.96 (0.92)   1. Pre: 21.69 (5.46)   Post: 18.30 (6.77)   1. Pre: 31.40 (8.56)   Post: 37.17 (9.86)   1. Pre: 0.31 (0.53)   Post: 0.39 (1.29)   1. Pre: 0.12 (0.22)   Post: 0.09 (0.21)   1. Pre: 1.36 (2.64)   Post: 0.78 (2.29) | 1. *F*=95.97, *p*<.001, *d*=1.37 2. *F*=108.22, *p*<.001, *d*=1.62 3. *F*=151.26, *p*<.001, *d*=1.68 4. *F*=16.50, *p*<.001, *d*=0.53 5. *F*=21.35, *p*<.001, *d*=1.03 6. *F*=48.88, *p*<.001, *d*=1.07 7. *F*=0.96, *p*=.327, *d*=.13 8. *F*=5.65, *p*=.018, *d*=.31 9. *F*=13.33, *p*<.001, *d*=.47 | Adolescents experienced significant improvements in their mental health symptomology and behaviour, and significant reductions in suicidality, conduct problems, and average monthly hospital admissions post-program. However, there was an increase in average monthly emergency department presentations. |
| Howe (2017) | Time 1: admission  Time 2: discharge | 1. Mental health clinical symptomology and functioning: HoNOSCA 2. General functioning: CGAS | 1. Pre: 15.42 (6.18)   Post: 7.52 (5.75)   1. Pre: 57.91 (10.40)   Post: 71.89 (12.51) | 1. *p*<.001, *d*=1.32 2. *p*<.001, *d*=1.22 | There was significant improvements in mental health clinical symptomology and general functioning following engagement in the service. |
| Killackey (2017) | Time 1: baseline  Time 2: post-intervention (6 months) | 1. Engagement in education 2. Level of education achieved | 1. 18 of 19 participants were engaged in education 2. 18 of 19 participants were studying beyond their previous highest level of education   Mean and SD Not reported | Significance and effect sizes not reported and cannot be calculated | 95% of participants were engaged in education, studying beyond their previous highest level of education after engaging in the program. |
| Killackey (2019) | Time 1: baseline  Time 2: post intervention (6 months)  Time 3: 12 months  Time 4: 18 months | 1. Employment status 2. Average hours worked 3. Studying status | 1. Intervention: 71%   Control: 48%   1. Not reported 2. Not reported | 1. *OR*=3.40, *p*=.025 2. *F*=0.95, *p*=.390 3. *OR*=0.87, *p*=.018   Effect sizes not reported and cannot be calculated | The intervention group had significantly greater odds of being in employment after 6 months, but no significant differences after 12 or 18 months for employment. No difference in hours worked. Greater odds of studying post-program. |
| Sabbioni (2018) | Time 1: first session  Time 2: fifth session  Time 3: last session | 1. Psychosocial wellbeing: Outcome Rating Scale 2. Mental health functioning: HoNOS | 1. Pre: 19.7 (9.15)   Post: 27 (10.5)   1. Pre: 17.3 (6.4)   Post: 14.4 (6.7) | 1. *t*(39), 4.0, *p*<.001, *d*= 0.69 2. Not recorded | Young people reported significant improvements in psychosocial wellbeing (personal, relational, social and global wellbeing) following engagement in YouthLink |
| Schley (2012) | Time 1: at referral  Time 2: following assessment  Time 3: discharge | 1. Overall treatment engagement: 11-item engagement measure 2. Suicidality: BPRS 3. Hostility: BPRS 4. Mental health functioning: HoNOSCA 5. Psychosocial functioning: Global Assessment of Functioning Scale | 1. Pre: 35.5 (10.4)   Post: 34.9 (12.2)   1. Pre: 2.7 (1.3)   Post: 2.1 (1.3)   1. Pre: 2.8 (1.7)   Post: 2.4 (1.5)   1. Pre: 1.5 (0.4)   Post: 1.0 (0.6)   1. Pre: 44.6 (13.1)   Post: 55.4 (15.3) | 1. *p*>.05, *d*=.05 2. *p*<.01, *d*=.46 3. *p*<.05, *d*=.25 4. *p*<.01, *d*=.98 5. *p*<.001, *d*=.76 | There was a significant reduction in suicidality and hostility, improved mental health and psychosocial functioning. |
| **Integrated ‘one-stop-shop’ Programs** | | | | | |
| **First Author (Year)** | **Assessment Time Points** | **Outcomes and Measures** | **Descriptive Statistics**^1^  **(M and SD)** | **Significance of Difference** | **Summary of Findings** |
| Bassilios (2017) | Time 1: pre-treatment  Time 2: post-treatment | 1. Psychological distress: K-10 | 1. Intervention: 23 (8.1)   Control: 22.6 (8.9) | 1. *p*>.05, *d*=.047 | There were no significant differences in the reduction of psychological distress following treatment between youth engaged in Headspace compared to Allied Psychological Services. |
| Goel (2021) | Time 1: admission  Time 2: discharge | 1. Mental health functioning: HoNOS 2. Psychological distress: K-10 3. Mental health-related inpatient admissions: % | 1. Pre: 11.4 (4.9)   Post: 6.3 (4.2)   1. Pre: 30.9   Post: 21.3  (SD not reported)   1. 7% | 1. *t*=12.2, *p*<.001, *d*=1.12 2. *p*<.01, effect size not reported and cannot be calculated 3. Not applicable | Youth who engaged in the Community Assessment and Treatment Service experienced significant reductions in mental health difficulties and psychological distress, with 93% avoiding hospital. |
| Green (2015) | Time 1: at referral  Time 2: discharge | 1. Body mass index (BMI) 2. Drive for thinness: Eating Disorder Inventory, third edition (EDI-III) 3. Body dissatisfaction: EDI-III 4. Bulimia: EDI-III 5. Depressive symptoms: The beck Depression Inventory-2 (BDI-II) 6. Anxiety symptoms: The Beck Anxiety Inventory (BAI) 7. Mental health functioning: HoNOSCA   Mental health functioning: HoNOS   1. General functioning: CGAS | Not reported | 1. *p*<.01 2. *p*<.001 3. *p*<.001 4. *p*>.05 5. *p*<.001 6. *p*<.001 7. *p*<.001 8. *p*<.05 9. *p*<.001   Effect sizes not reported and cannot be calculated | A longer duration of time spent in the program yielded a significant increase in BMI and general functioning, while drive for thinness, body dissatisfaction, anxiety, depression, and general mental health symptoms significantly decreased. |
| Kennair (2011) | Time 1: one week post program commencement  Time 2: one week post program completion | 1. Mental health functioning: HoNOSCA | Not recorded | 1. *F*(1, 165) = 4.89, *p*<.05, *η^2^* = .03 | Participants experienced a significant improvement in mental health functioning following engagement in the Adolescent Day Program. |
| Klag (2021) | Time 1: at referral  Time 2: post-program (average of 19 months) | 1. Mental health symptoms: HoNOSCA 2. General functioning: CGAS | 1. Overall mean and SD not reported 2. Pre: 48.2   Post: 57.8  SD not reported | 1. p<.05 for 10 of 13 items 2. *t*=12.6, *p*<.001   Effect sizes not reported and cannot be calculated | A statistically significant number of young people moved from ‘clinical’ to ‘non-clinical’ from pre to post treatment for 11 of 13 HoNOSCA items. There was also a significant improvement in overall functioning. |
| Rickwood (2015) | Time 1: first contact  Time 2: final session | 1. Psychological distress: K-10 2. Psychosocial functioning: Social and Occupational Functioning Assessment Scale | Not recorded | 1. *p*<.05 2. *p<*.05   Effect sizes not reported and cannot be calculated | Headspace clients experienced reduced psychological distress and improved psychosocial functioning after receiving support. |
| **Lifestyle Programs** | | | | | |
| **First Author (Year)** | **Assessment Time Points** | **Outcomes and Measures** | **Descriptive Statistics**^1^  **(M and SD)** | **Significance of Difference** | **Summary of Findings** |
| Curtis (2016) | Time 1: referral  Time 2: post-intervention (12 weeks) | 1. Weight 2. Body mass index 3. Psychosocial functioning: Global assessment of functioning 4. Mental health functioning: HoNOS 5. Sleep quality: Pittsburgh Sleep Quality Index 6. Self-esteem: Rosenberg Self-Esteem Scale 7. Antipsychotic medication adherence: Medication Adherence Rating Scale 8. Energy consumption (kcal/day): Food frequency questionnaire 9. Physical activity (min/week): International Physical Activity Questionnaire-Short Form | 1. Intervention: 69.9 (13.9)   Control: 83.6 (19.0)   1. Intervention: 23.9 (3.2)   Control: 27.4 (3.1)   1. Pre: 54.6 (7.8)   Post: 70.7 (9.9)   1. Pre: 9.9 (3.4)   Post: 6.1 (5.4)   1. Pre: 7.7 (3.6)   Post: 6.1 (2.3)   1. Pre: 18.4 (6.9)   Post: 19.1 (5.4)   1. Pre: 7.9 (1.9)   Post: 8.3 (1.8)   1. Pre: 2015.7 (535.8)   Post: 1507.8 (471.5)   1. Pre: 788.4 (655.0)   Post: 2027.6 (1740.0) | 1. *F*(1, 25) = 19.6, *p*<.001, *d*=.82 2. *F*(1, 25) = 23.3, *p*<.001, *d*=1.11 3. *t*(15) = 9.0, *p*<.001, *d*=1.81 4. *t*(15) = 2.5, *p*<.05, *d*=.84 5. *t*(15) = 2.3, *p*<.05, *d*=.53 6. *t*(15) = 0.7, *p*>.05, *d*=.11 7. *t*(15) = 0.3, *p*>.05, *d*=.22 8. *t*(15) = 4.4, *p*<.001, *d*=1.01 9. *t*(15) = 2.7, *p*<.05, *d*=.94 | There was a significant reduction in weight and BMI, improved mental health, psychosocial functioning and sleep, and increased energy consumption and physical activity post-intervention. |
| McGuire (2021) | Time 1: pre intervention  Time: post intervention (6 weeks) | Anxiety-related outcomes were measured using The State-Trait Anxiety Inventory-6 (STAI-6):   1. Calmness 2. Relaxation 3. Content 4. Tense 5. Upset 6. Worry | SD not reported   1. Pre: 2.66   Post: 3.28   1. Pre: 2.61   Post: 3.46   1. Pre: 2.50   Post: 3.07   1. Pre: 2.21   Post: 1.59   1. Pre: 1.76   Post: 1.34   1. Pre: 1.82   Post: 1.54 | Effect sizes not reported and cannot be calculated   1. *t*=2.768, *p*=.01 2. *t*=5.645, *p*<.001 3. *t*=3.286, *p*=.003 4. *t*=4.312, *p*<.001 5. *t*=2.703, *p*<.012 6. *t*=1.867, *p*=.073 | Young people reported feeling significantly calmer, relaxed, content, and less tense and upset following the yoga intervention. The reduction in worry was not statistically significant. |
| Nasstasia (2017) | Baseline, weeks 2, 4, 5, 6, 8, 10, 12 (post-intervention), and 9 months after baseline | 1. Diagnosis of Major Depressive Disorder: the % of participants who no longer met criteria using the Structured Clinical Interview for DSM-IV-TR Disorders (SCID) 2. Behavioural activation: The Behavioural Activation for Depression Scale-Short Form (BADS-SF) 3. Depressive symptoms: The Beck Depression Inventory (BDI-II) 4. Negative thoughts: The Automatic Thoughts Questionnaire (ATQ) | 1. Pre-intervention: 0%   Post-intervention: 75%  Mean change in scores:   1. 1.49 (1.43) 2. -1.79 (1.17) 3. -1.59 (1.45) | 1. Not reported   Effect sizes not reported and cannot be calculated   1. *p*<.01 2. *p*<.01 3. *p*<.05 | After receiving the motivational interviewing and exercise intervention, participants had significantly reduced depressive symptoms and increased behavioural activation, with 75% no longer meeting criteria for major depressive disorder. |
| Nasstasia (2019) | Time 1: at referral  Time 2: mid-treatment (6 weeks)  Time 3: post-intervention (12 weeks)  Time 4: 24 weeks follow-up (12 weeks post-intervention) | 1. Diagnosis of Major Depressive Disorder: the % of participants who no longer met DSM-IV criteria using the SCID 2. Depressive symptoms: BDI 3. Somatic symptoms: Depression Somatic Symptom Scale 4. Self-esteem: Single Item Self-Esteem Questionnaire | 1. Intervention: 62%   Control: 10%   1. Intervention: 14.91 (8.90)   Control: 28.37 (8.84)   1. Intervention: 2.87 (3.65)   Control: 8.33 (5.14)   1. Intervention: 2.77 (0.97)   Control: 2.07 (0.83) | 1. Not recorded 2. *W^2^*=14.59, *p*<.001, *d*=1.52 3. *W^2^*=8.64, *p*<.01, *d*=1.22 4. *W^2^*=5.69, *p*<.05, *d*=.78 | Youth experienced improved self-esteem, and significant reductions in depressive and somatic symptoms, leading to 62% of the sample no longer meeting criteria for major depressive disorder, in comparison to 10% among controls. |
| Parker (2016) | Time 1: pre-treatment  Time 2: post-treatment (6 weeks) | 1. Depressive symptoms: BDI 2. Anxiety symptoms: Beck Anxiety Inventory 3. Substance use: Substance and Choices Scale 4. Psychosocial functioning: Social and Occupational Functioning Assessment Scale 5. Physical activity engagement: Days spent engaged in intensity and duration of activity that met the guideline recommendations | 1. Intervention: 9.50 (1.28)   Control: 14.09 (1.28)   1. Intervention: 6.09 (1.05)   Control: 7.88 (1.06)   1. Not recorded 2. Not recorded 3. Pre: 0.73 (1.21)   Post: 1.47 (1.81)  Standard error reported instead of SD | 1. *t*(136.6)=2.30, *p=*.023 2. *p*=.584 3. *p*>.05 4. *p*=3.74 5. *p*>.05   Effect sizes not reported and cannot be calculated | There were significant reductions in depressive symptoms among those in the physical activity intervention compared to the comparison group, but no significant differences in other outcomes. |
| Pearce (2020) | Time 1: upon referral  Time 2: upon discharge | 1. Weight (kg) 2. Proportion of sample classified as ‘overweight’ or ‘obese’ | 1. Pre: 86.3 (27.2)   Post: 90.0 (27.6)   1. Pre: 64%   Post: 72% | 1. *p*<.001, *d*=.14 2. *p*<.001 | There were significant increases in the weight and proportion of young people classified as overweight or obese at follow-up. |

*Note.* NR = Not Reported; NA = Not Applicable; M = Mean; SD = Standard Deviation.

^1^For controlled studies, data is presented for the time-point immediately following the intervention (post). For pre-post studies, data is presented for the first assessment time-point (pre) and immediately following the intervention (post), as only a minority of studies included an extended follow-up time-point.

Supplementary Table 4. *Quality Assessment of Controlled Intervention Studies*

| Criteria | First Author (Year) | | | | | |
| --- | --- | --- | --- | --- | --- | --- |
|  | Bassilios (2017) | Curtis (2016) | Havighurst (2015) | Hayes (2011) | Hides (2011) | Kennair (2011) |
| 1. Was the study described as a randomised trial, randomised clinical trial, or an RCT? | N | N | Y | Y | N | N |
| 2. Was the method of randomisation adequate (i.e., use of randomly generated assignment)? | NA | NA | Y | Y | NA | NA |
| 3. Was the treatment allocation concealed (so that assignments could not be predicted)? | NA | NA | Y | N | NA | NA |
| 4. Were study participants and providers blinded to treatment group assignment? | N | N | N | N | N | N |
| 5. Were the people assessing the outcomes blinded to the participants' group assignments? | N | NR | N | N | Y | N |
| 6. Were the groups similar at baseline on important characteristics that could affect outcomes (e.g., demographics, risk factors, co-morbid conditions)? | N | Y | N | Y | Y | Y |
| 7. Was the overall drop-out rate from the study at endpoint 20% or lower of the number allocated to treatment? | Y | N | Y | N | N | Y |
| 8. Was the differential drop-out rate (between treatment groups) at endpoint 15 percentage points or lower? | Y | Y | Y | Y | Y | Y |
| 9. Was there high adherence to intervention protocols for each treatment group? | NR | NR | Y | NR | N | NR |
| 10. Were other interventions avoided/similar in the groups (e.g., similar background treatments)? | Y | Y | Y | N | N | Y |
| 11. Were outcomes assessed using valid and reliable measures, implemented consistently across all study participants? | Y | Y | Y | Y | Y | Y |
| 12. Was the sample size sufficiently large to detect a difference in outcomes between groups with at least 80% power? | N | N | Y | N | N | Y |
| 13. Were outcomes reported or subgroups analysed prespecified (i.e., identified before analyses were conducted)? | Y | Y | Y | Y | Y | Y |
| 14. Were all randomised participants analysed in the group they were originally assigned? (i.e., use of intention-to-treat analysis)? | Y | Y | Y | Y | Y | Y |
| Total Score (maximum = 14) | 6 | 6 | 11 | 7 | 6 | 8 |
| Risk of Bias | High | High | Low | Med | High | Med |
| Quality Assessment | Low | Low | High | Med | Low | Med |

*Note.* Y = Yes; N = No; NA = Not applicable; NR = Not reported; Med = medium.

| Criteria | First Author (Year) | | | | | | |
| --- | --- | --- | --- | --- | --- | --- | --- |
|  | Keyhoe (2014) | Killackey (2019) | Nasstasia (2019) | Parker (2016) | Poole (2018) | Tan (2015) | Wagner (2017) |
| 1. Was the study described as a randomised trial, randomised clinical trial, or an RCT? | Y | Y | Y | Y | Y | Y | N |
| 2. Was the method of randomisation adequate (i.e., use of randomly generated assignment)? | Y | Y | Y | Y | Y | NR | NA |
| 3. Was the treatment allocation concealed (so assignments could not be predicted)? | Y | Y | Y | Y | Y | NR | NA |
| 4. Were study participants and providers blinded to treatment group assignment? | N | N | N | N | Y | N | N |
| 5. Were the people assessing the outcomes blinded to participants' group assignments? | N | Y | Y | Y | Y | N | N |
| 6. Were the groups similar at baseline on important characteristics that could affect outcomes (e.g., demographics, risk factors, co-morbid conditions)? | Y | N | Y | Y | Y | Y | Y |
| 7. Was the overall drop-out rate from the study at endpoint 20% or lower of the number allocated to treatment? | N | Y | N | N | Y | Y | Y |
| 8. Was the differential drop-out rate (between treatment groups) at endpoint 15 percentage points or lower? | N | Y | Y | Y | Y | Y | Y |
| 9. Was there high adherence to intervention protocols for each treatment group? | Y | Y | N | NR | Y | NR | NR |
| 10. Were other interventions avoided/similar in the groups (e.g., similar background treatments)? | Y | Y | Y | Y | Y | Y | Y |
| 11. Were outcomes assessed using valid and reliable measures, implemented consistently across all study participants? | Y | N | Y | N | Y | N | Y |
| 12. Was the sample size sufficiently large to detect a difference in outcomes between groups with at least 80% power? | NR | Y | Y | Y | NR | Y | Y |
| 13. Were outcomes reported or subgroups analysed prespecified (i.e., identified before analyses were conducted)? | Y | Y | Y | Y | Y | Y | Y |
| 14. Were all randomised participants analysed in the group they were originally assigned? (i.e., intention-to-treat analysis)? | Y | Y | Y | Y | Y | Y | Y |
| Total Score (maximum = 14) | 9 | 11 | 11 | 10 | 13 | 8 | 8 |
| Risk of Bias | Med | Low | Low | Med | Low | Med | Med |
| Quality Assessment | Med | High | High | Med | High | Med | Med |

*Note.* Y = Yes; N = No; NA = Not applicable; NR = Not reported; Med = medium.

Supplementary Table 4. *Quality Assessment of Before-After (Pre-Post) Studies With No Control Group*

| Criteria | First Author (Year) | | | | | |
| --- | --- | --- | --- | --- | --- | --- |
|  | Brewer (2015) | Chia (2013) | Conrad (2017) | Daubney (2021) | Edwards (2018) | Farrell (2012) |
| 1. Was the study question or objective clearly stated? | Y | Y | Y | Y | Y | Y |
| 2. Were eligibility/selection criteria for the study population prespecified/clearly described? | Y | Y | N | Y | N | Y |
| 3. Were the participants in the study representative of those who would be eligible for the intervention in the general or clinical population of interest? | N | N | Y | Y | N | NR |
| 4. Were all eligible participants that met the prespecified entry criteria enrolled? | Y | Y | NR | Y | N | Y |
| 5. Was the sample size sufficiently large to provide confidence in the findings? | Y | NR | Y | N | N | N |
| 6. Was the intervention clearly described and delivered consistently across the study population? | Y | Y | NR | NR | Y | Y |
| 7. Were outcome measures prespecified, clearly defined, valid, reliable, and assessed consistently across all study participants? | Y | Y | Y | Y | N | Y |
| 8. Were the people assessing the outcomes blinded to the participants’ intervention? | N | NR | NR | N | N | NR |
| 9. Was the loss to follow-up after baseline 20% or less? Were those lost to follow-up accounted for in the analysis? | NR | Y | NR | Y | Y | Y |
| 10. Did the statistical methods examine changes in outcome measures from before to after the intervention? Did statistical tests provide p values for the pre-to-post change? | Y | Y | Y | Y | N | Y |
| 11. Were outcome measures of interest taken multiple times before the intervention and multiple times after the intervention (i.e., did they use an interrupted time-series design)? | N | N | Y | N | Y | N |
| 12. If the intervention was conducted at a group level (e.g., a whole hospital, a community), did the statistical analysis take into account the use of individual-level data to determine effects at the group level? | NA | NA | NA | NA | NA | NA |
| Total Score (maximum = 12) | 7 | 7 | 6 | 7 | 4 | 7 |
| Risk of Bias | Med | Med | Med | Med | High | Med |
| Quality Assessment | Med | Med | Med | Med | Low | Med |

*Note.* Y = Yes; N = No; NA = Not applicable; NR = Not reported; Med = medium.

| Criteria | First Author (Year) | | | | | |
| --- | --- | --- | --- | --- | --- | --- |
|  | Goel (2021) | Green (2015) | Hall (2021) | Hickey (2020) | Hopkins (2017) | Howe (2017) |
| 1. Was the study question or objective clearly stated? | Y | Y | Y | Y | Y | Y |
| 2. Were eligibility/selection criteria for the study population prespecified/clearly described? | Y | Y | N | Y | N | N |
| 3. Were the participants in the study representative of those who would be eligible for the intervention in the general or clinical population of interest? | N | N | Y | N | N | Y |
| 4. Were all eligible participants that met the prespecified entry criteria enrolled? | Y | N | NR | Y | NR | NR |
| 5. Was the sample size sufficiently large to provide confidence in the findings? | Y | N | N | N | N | Y |
| 6. Was the intervention clearly described and delivered consistently across the study population? | Y | Y | Y | Y | NR | Y |
| 7. Were outcome measures prespecified, clearly defined, valid, reliable, and assessed consistently across all study participants? | Y | Y | Y | Y | Y | N |
| 8. Were the people assessing the outcomes blinded to the participants’ intervention? | N | N | NR | N | N | Y |
| 9. Was the loss to follow-up after baseline 20% or less? Were those lost to follow-up accounted for in the analysis? | Y | Y | N | Y | Y | Y |
| 10. Did the statistical methods examine changes in outcome measures from before to after the intervention? Did statistical tests provide p values for the pre-to-post change? | Y | Y | Y | Y | Y | Y |
| 11. Were outcome measures of interest taken multiple times before the intervention and multiple times after the intervention (i.e., did they use an interrupted time-series design)? | N | Y | N | Y | N | N |
| 12. If the intervention was conducted at a group level (e.g., a whole hospital, a community), did the statistical analysis take into account the use of individual-level data to determine effects at the group level? | NA | NA | NA | NA | NA | NA |
| Total Score (maximum = 12) | 8 | 7 | 5 | 8 | 4 | 7 |
| Risk of Bias | Med | Med | High | Med | High | Med |
| Quality Assessment | Med | Med | Low | Med | Low | Med |

*Note.* Y = Yes; N = No; NA = Not applicable; NR = Not reported; Med = medium.

| Criteria | First Author (Year) | | | | | |
| --- | --- | --- | --- | --- | --- | --- |
|  | Hudson (2015) | Killackey (2017) | Klag (2021) | McGuire (2021) | Nasstasia (2017) | Pearce (2020) |
| 1. Was the study question or objective clearly stated? | Y | Y | Y | Y | Y | Y |
| 2. Were eligibility/selection criteria for the study population prespecified/ clearly described? | Y | Y | Y | Y | Y | Y |
| 3. Were the participants in the study representative of those who would be eligible for the intervention in the general or clinical population of interest? | Y | N | Y | N | N | Y |
| 4. Were all eligible participants that met the prespecified entry criteria enrolled? | Y | Y | Y | NR | N | Y |
| 5. Was the sample size sufficiently large to provide confidence in the findings? | Y | N | Y | N | N | Y |
| 6. Was the intervention clearly described and delivered consistently across the study population? | Y | Y | Y | N | Y | Y |
| 7. Were outcome measures prespecified, clearly defined, valid, reliable, and assessed consistently across all study participants? | Y | Y | Y | Y | Y | Y |
| 8. Were the people assessing the outcomes blinded to the participants’ intervention? | N | N | N | N | N | N |
| 9. Was the loss to follow-up after baseline 20% or less? Were those lost to follow-up accounted for in the analysis? | N | Y | Y | Y | N | Y |
| 10. Did the statistical methods examine changes in outcome measures from before to after the intervention? Did statistical tests provide p values for the pre-to-post change? | Y | N | Y | Y | Y | Y |
| 11. Were outcome measures of interest taken multiple times before the intervention and multiple times after the intervention (i.e., did they use an interrupted time-series design)? | N | N | N | N | Y | N |
| 12. If the intervention was conducted at a group level (e.g., a whole hospital, a community), did the statistical analysis take into account the use of individual-level data to determine effects at the group level? | NA | NA | NA | NA | NA | NA |
| Total Score (maximum = 12) | 8 | 6 | 9 | 5 | 6 | 9 |
| Risk of Bias | Med | Med | Low | High | Med | Low |
| Quality Assessment | Med | Med | High | Low | Med | High |

*Note.* Y = Yes; N = No; NA = Not applicable; NR = Not reported; Med = medium.

| Criteria | First Author (Year) | | | | | |
| --- | --- | --- | --- | --- | --- | --- |
|  | Porter (2016) | Rickwood (2015) | Sabbioni (2018) | Schley (2012) | Schley (2018) | Westwater (2020) |
| 1. Was the study question or objective clearly stated? | Y | Y | Y | Y | Y | Y |
| 2. Were eligibility/selection criteria for the study population prespecified/clearly described? | Y | Y | N | N | N | Y |
| 3. Were the participants in the study representative of those who would be eligible for the intervention in the general or clinical population of interest? | Y | Y | Y | Y | Y | N |
| 4. Were all eligible participants that met the prespecified entry criteria enrolled? | Y | N | NR | N | Y | N |
| 5. Was the sample size sufficiently large to provide confidence in the findings? | Y | Y | NR | Y | N | N |
| 6. Was the intervention clearly described and delivered consistently across the study population? | Y | NR | Y | Y | N | N |
| 7. Were outcome measures prespecified, clearly defined, valid, reliable, and assessed consistently across all study participants? | Y | Y | Y | Y | Y | Y |
| 8. Were the people assessing the outcomes blinded to the participants’ interventions? | N | N | N | N | N | N |
| 9. Was the loss to follow-up after baseline 20% or less? Were those lost to follow-up accounted for in the analysis? | N | N | Y | Y | N | N |
| 10. Did the statistical methods examine changes in outcome measures from before to after the intervention? Did statistical tests provide p values for the pre-to-post change? | Y | Y | Y | Y | Y | Y |
| 11. Were outcome measures of interest taken multiple times before the intervention and multiple times after the intervention (i.e., did they use an interrupted time-series design)? | Y | Y | N | Y | N | N |
| 12. If the intervention was conducted at a group level (e.g., a whole hospital, a community), did the statistical analysis take into account the use of individual-level data to determine effects at the group level? | NA | NA | NA | NA | NA | NA |
| Total Score (maximum = 12) | 9 | 7 | 6 | 8 | 5 | 4 |
| Risk of Bias | Low | Med | Med | Med | High | High |
| Quality Assessment | High | Med | Med | Med | Low | Low |

*Note.* Y = Yes; N = No; NA = Not applicable; NR = Not reported; Med = medium.
